# Rate of Entropy Production in Evolving Interfaces and Membranes under Astigmatic Kinematics: Shape Evolution in Geometric-Dissipation Landscapes

**DOI:** 10.3390/e22090909

**Published:** 2020-08-19

**Authors:** Ziheng Wang, Phillip Servio, Alejandro D. Rey

**Affiliations:** Department of Chemical Engineering, McGill University, 3610 University Street, Montreal, QC H3A 2B2, Canada; ziheng.wang@mail.mcgill.ca (Z.W.); phillip.servio@mcgill.ca (P.S.)

**Keywords:** entropy production rate, Boussinesq-Scriven dissipation, surface evolution, astigmatic flow, shape parameter, Casorati curvature, pure growth

## Abstract

This paper presents theory and simulation of viscous dissipation in evolving interfaces and membranes under kinematic conditions, known as astigmatic flow, ubiquitous during growth processes in nature. The essential aim is to characterize and explain the underlying connections between curvedness and shape evolution and the rate of entropy production due to viscous bending and torsion rates. The membrane dissipation model used here is known as the Boussinesq-Scriven fluid model. Since the standard approaches in morphological evolution are based on the average, Gaussian and deviatoric curvatures, which comingle shape with curvedness, this paper introduces a novel decoupled approach whereby shape is independent of curvedness. In this curvedness-shape landscape, the entropy production surface under constant homogeneous normal velocity decays with growth but oscillates with shape changes. Saddles and spheres are minima while cylindrical patches are maxima. The astigmatic flow trajectories on the entropy production surface, show that only cylinders and spheres grow under the constant shape. Small deviations from cylindrical shapes evolve towards spheres or saddles depending on the initial condition, where dissipation rates decrease. Taken together the results and analysis provide novel and significant relations between shape evolution and viscous dissipation in deforming viscous membrane and surfaces.

## 1. Introduction

The shape of surfaces, interfaces and membranes can be efficiently described by a normalized scalar dimensionless shape parameter (*S*) that discriminates between patches of spheres, cylinders, saddles and intermediate states [1]. Likewise, surface curvedness (*C*) or deviation from planarity, can be characterized by a positive scalar (the condition reduces to a plane if C=0), with natural units of inverse length. Shape parameter *S* and curvedness *C* are essential features and determinants of surface properties and functionalities that can be leveraged in many engineering [2,3], biological and biomimetic applications [4,5,6,7,8].

The influence of these intrinsic geometric attributes (C,S) can be seen in interfacial transport and rheology [9], the preferred configuration of surfaces for proteins [10] and in dendritic microstructures during coarsening [11,12], to name a few. Shape and curvedness affect membrane elasticity [13], the rate of growth of cell tissues [14] and particle behaviours [15]. For example, the shape and curvedness of liquid crystals are sensitive to external stimuli such as electromagnetic field [16], light stimulation [17,18], force field [19], surface anchoring [20,21,22,23,24,25,26,27] and surfactants [28,29]. They can be applied in superhydrophobic materials [30] and optical lenses [31,32].

An important aspect in equilibrium and non-equilibrium self-assembly, growth, phase ordering transitions, phase separation, accretion, abrasion and morphogenesis is the spatio-temporal evolution of shape and curvedness as controlled by kinematics and dynamics [33]. Since interfacial and membrane dissipation during evolving shape and curvedness [1] involves rates of change of bending and torsion, there is a natural and direct connection between entropy production rates and time-dependent geometric variations. The connection between geometry and thermodynamics has been long studied mainly in equilibrium and statics. For example, curvatures serve as potential barriers in matter aggregation [34], in biomolecular aggregation [35], or the growing analysis based on the diffusive-convective fields [36] and Vojta–Natanson principle [37], other examples can be found in [38,39,40].

In this work, we initiate a similar framework but for dissipative evolution and growth, seeking to establish how the rate of entropy production manifests in shape and curvedness temporal changes, say, from a highly curved cylindrical patch to a flatter saddle patch. To maintain a realistic scope for the paper, we only consider surface patches, as opposed to closed shapes, and investigate viscous dissipation in moving and deforming surfaces, interfaces and membranes under given constant growth kinematics, where the velocity has only a normal component hence the surface curvatures are simply related to each other, a condition known as astigmatic flow since it corresponds to surfaces whose time-dependent curvatures are related to each other by a constant [1]. Other surface kinematics, such as mean curvature flow or Ricci flow are not treated in this work.

To achieve the essential goal of this paper, that is to establish and characterize the connection between evolving surfaces with changing shapes and curvedness with the rate of entropy production, we formulate a novel method that expresses the rate of dissipation due to torsion and bending rates in terms of the actual shape and curvedness conditions. In this approach, the entropy production rate surface is defined as a Monge surface patch [41] with shape parameter *S* and curvedness *C* as coordinates. The resulting entropy production surface geometry (curvatures, main directions, metric, critical points) provides new insights on non-equilibrium morphogenesis.

**Figure 1:**
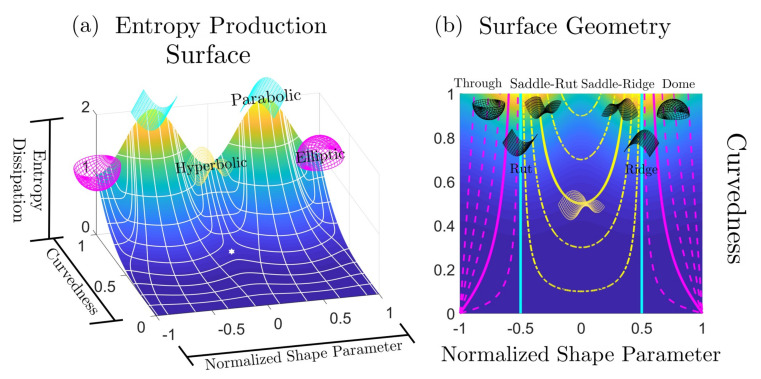
Schematic visualization of the concepts, procedures, and objectives of this paper on the relation between entropy production Δ in evolving surfaces (**a**) under constant normal velocity (astigmatic flow), (**b**) using a novel shape (*S*)-curvedness (*C*) framework. (**a**) We construct an entropy production surface (Monge patch) using the Boussinesq-Scriven dissipation model, where each set (C,S) defines a parabolic, elliptic or hyperbolic physical surface and determines the important geometric features such as lines of curvature (LOC: white lines), and umbilic points (defect: white dot). (**b**) Physical surface geometry in the (C,S)-frame. Growth and evolution are defined by lines on the curvedness-shape plane, where following the magenta, cyan and yellow lines the surface evolves to a sphere, cylinder or saddle, respectively. These growth-evolution lines show how shape changes (magenta or yellow) or remains intact (cyan). The final step and goal are to embed and study how these astigmatic flow lines shown in (**b**) correspond to varying entropy production landscape shown in (**a**), and to derive evolving surface geometry-entropy production scaling relations.

Figure 1 shows a schematic that summarizes the key objectives, the technical concepts, workflow, and the nomenclature used in the paper. To achieve the characterization of rates of entropy production due to evolving physical surface geometry under astigmatic flow we perform the following workflow steps:

1. Using the well-known Boussinesq-Scriven dissipation [1,42,43] due to bending and torsion rates, we reformulate this quantity in terms of curvedness and shape. This is done by expressing bending and twisting rates in terms of actual curvedness and shape and not their rates. This is a crucial step in the present approach. The result of this initial step, shown on Figure 1a, can be expressed in terms of a Monge surface patch [41] Δ(C,S) for the entropy production rate in terms of its natural coordinates: shape (*S*), and curvedness (*C*);
2. We then characterize the geometry of this entropy production surface Δ(C,S) including its curvatures, geodesics, metric, critical points, principal lines of curvature, lines of steepest descent and level set curves [44]. The lines of curvature and umbilical (defect) points are revealed by Figure 1a. This is an essential step to capture the geometry of the rate of entropy production surface;
3. Each point on the thermodynamic surface Δ(C,S) corresponds to a physical surface shape (sphere, cylinder, saddle) by fixing curvedness, creating an opportunity to establish a direct connection between the physical surface shape and dissipation rate, as follows from Figure 1. The top three surface patches (magenta: sphere, cyan: cylinder and yellow: saddle) of Figure 1a show characteristic shapes of elliptic, parabolic and hyperbolic patches, respectively. Figure 1b shows the projection of Figure 1a on the (C,S)-frame. The growth of a surface patch uniquely changes its shape and curvedness, exhibiting as a curve on the (C,S)-frame. This curve is known as the astigmatic flow. Figure 1b shows a series of astigmatic flow curves. For example, the yellow line shows a hyperbolic patch (saddle-rut or saddle-ridge) that changes into a perfect saddle (S=0). Following the magenta line, an elliptic patch (through or dome) changes into a more spherical patch. We note that the C=0 line is a flat surface with no shape. The nomenclature in Figure 1b which distinguish the sign of the shape are more commonly used in engineering;
4. As this work only considers astigmatic flow [1], we establish and study the evolution lines in detail, given by the astigmatic flow: C=f(S,m), where *m* is an invariant that defines a particular shape evolution trajectory. The evolutions are planar but curved lines on the thermodynamic surface under the (C,S)-frame. Of particular interest are cases of constant shape evolutions, which are only found for growing spherical and cylindrical patches and serve as important special reference cases;
5. The final step is to integrate steps 1–4. Studying these geometric astigmatic trajectories on the entropy production rate landscape we evaluate when the evolution samples high and low entropy production rates, and ultimately establish the corresponding scaling laws.

The rate of entropy production is given by the generalized Boussinesq-Scriven dissipation rate [45]. In this paper, we only consider the entropy production due to the shape and curvedness change under a particular given kinematics (see Equation (10)). Other cases and other sources of entropy production are beyond the scope of this paper. The frame of the rate of entropy production surfaces is the natural (C,S)-frame. The entropy production surface is given by a Monge patch [41]. Heat dissipation, in-plane deformation rates (dilation and shear), tangential flows, non-constant normal velocity, and non-local effects are not considered.

In order to solve this problem, we propose a method that incorporates the rate of entropy production with the language of decoupled shape and curvedness parameters. In this paper, (1) the rate of entropy production as a function of shape and cruvedness is presented; (2) geometric properties of the surface of rate of entropy production are evaluated; (3) the astigmatic flow, which is one of the most common surface evolving kinematic, is analyzed by projecting on the surface of entropy production rate.

This paper is organized as indicated in the five steps given above. First, a brief background of surface evolution is fully discussed in Section 2, including how to describe local surface geometry by two independent parameters: curvedness *C* and shape parameter *S* (Section 2.1), how to describe astigmatic flows in the (C,S)-frame (Section 2.2), and how to quantify the Boussinesq-Scriven dissipation [1] of this process (Section 2.3). The results of the entropy dissipation Δ in the (C,S)-frame for the astigmatic kinematics are evaluated in Section 3. The results are divided into three sections: (1) The geometric evolution (Section 3.1) of the astigmatic flow (Section 3.1.1) and the rate of curvatures change (Section 3.1.2); (2) The thermodynamic surface Δ (Section 3.2), including its decomposition into primary shape and curvedness contributions (Section 3.2.1), the geometric properties such as lines of curvature (Section 3.2.2) and geodesics (Section 3.2.3); (3) The integration of the physical surface (Section 3.3), where we discuss how Δ behaves following the astigmatic flow (Section 3.3.1), the relationship between the astigmatic flow of and the geometric flow (Section 3.3.2), as well as the average entropy production rate (Section 3.3.3). The summary and conclusion of this paper are given in Section 4. The mathematical derivation details of the most important equations are given in the appendices. For brevity and without ambiguity, in most of the text, surface means a surface, an interface or a membrane.

## 2. Background of Surface Evolution Model

### 2.1. Local Geometry Description by the Shape-Curvedness Method

Any 2×2 surface tensor can be decomposed using the four fundamental surface tensor bases (see Appendix A), facilitating shape and curvedness derivations; here $I_{s}$ is the unit dyadic, q the symmetric deviatoric dyadic, $q_{1}$ the symmetric off-diagonal dyadic and $\epsilon_{s})$ is the surface alternator tensor [1]. Below we use these four surface tensor bases to express various curvature tensors that arise in the present model.

The 2×2 symmetric curvature tensor b and the surface gradient of the surface unit normal k add to zero [1]:

(1) $$b=-\nabla_{s}k=\kappa_{1}e_{1}e_{1}+\kappa_{2}e_{2}e_{2}$$

where $\kappa_{i}$ and $e_{i}$ are the eigenvalues (principal curvatures) and eigenvectors of the curvature tensor. $\nabla_{s}=I_{s}\cdot \nabla$ is the surface gradient operator. The symmetric curvature tensor b can be decomposed into a trace curvature tensor $HI_{s}$ and deviatoric curvature tensor $Dq=b-HI_{s}:=-f$ such that $b=HI_{s}+Dq$. The Gaussian curvature is defined by $K=\kappa_{1}\kappa_{2}$, the mean curvature by $H=(\kappa_{1}+\kappa_{2})/2$ (in the following content we assume the principal curvatures $\kappa_{1}\geq \kappa_{2}$), and the deviatoric curvature by $D=(\kappa_{1}-\kappa_{2})/2>0$. The Casorati curvature *C* [1] measures the magnitude of the curvature. These basic quantities are summarized in Table 1 in terms of the curvature tensor decomposition and principal curvatures $\left(\kappa_{1},\kappa_{2}\right)$.

Since all the common curvatures (K,D,H) given in Table 1 have units, they necessarily comingle information on the shape (dimensionless) and curvedness, which are not efficient and appropriate to describe viscous dissipation by shape evolution. To work with a dimensionless shape measure, that can discriminate primitive forms such as spheres, cylinders and saddles, another formulation is needed. In this paper, we use the shape parameter, whose normalized form *S* (−1≤S≤1) is a dimensionless scalar defined by [1,46]

(2) $$S=\frac{2}{\pi }\arctan\left(\frac{H}{D}\right)$$

The sign of *S* is consistent with the mean curvature *H*. From Table 1 and Equation (2) the following relationships between the classical curvatures (H,D,K) and the shape-curvedness (S,C) quantities are found by

(3) $$H=C\sin\left(\frac{S\pi }{2}\right),\;D=C\cos\left(\frac{S\pi }{2}\right)\;\mathrm{and}\;K=-C^{2}\cos S\pi$$

Equation (3) verifies that the information of shape and curvedness are coupled in all the classical curvatures (H,D,K). The classical curvature descriptor (H,D)-frame and the novel (C,S)-frame are related by the Jacobian matrix J (detJ≠0):

(4) $$\dot{D}:=\left[\begin{array}{c} \dot{H}\\ \dot{D} \end{array}\right]=\left[\begin{array}{cc} \sin(\frac{S\pi }{2})\; & \frac{\pi }{2}C\cos\left(\frac{S\pi }{2}\right)\\ \cos(\frac{S\pi }{2})\; & -\frac{\pi }{2}C\sin\left(\frac{S\pi }{2}\right) \end{array}\right]\cdot \left[\begin{array}{c} \dot{C}\\ \dot{S} \end{array}\right]=J\cdot \dot{S}$$

where we denote D as the ${\left[\begin{array}{cc} H & D \end{array}\right]}^{⊺}$ and S as the ${\left[\begin{array}{cc} C & S \end{array}\right]}^{⊺}$ vector for simplicity, and a superdot for the time derivative.

**Figure 2:**
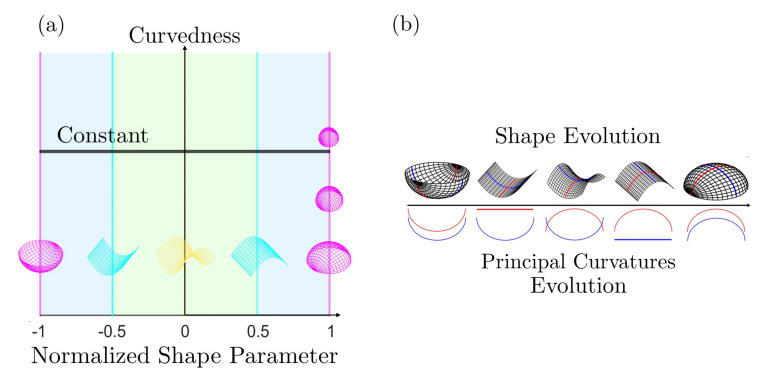
(**a**) The shape (*S*)-curvedness (*C*) frame. Each point on this plane corresponds to a surface patch characterized by these two values. The curvedness remains the same along the constant $C_{0}$ line (horizontal), and the shape is invariant along a vertical line. The shape symmetry is captured by the sign of *S*: concave-up (S<0) and concave-down (S>0). (**b**) The shape and principal curvatures evolution along the direction of increasing *S* and constant *C*, where blue and red curves represent two circles with radii of the reciprocal of the principal curvatures. The top of Figure 1b defines the terminology for intermediate surface shapes used in applications, while Figure 2b shows the primary shapes: spheres (two ends), saddle (centre) and cylinders (in-between).

Figure 2a shows the distribution of different surface patches in the (C,S)-frame; *S* varies from −1 to +1 and *C* varies from 0 (*S*-axis) to plus infinity. Recall that the surface is flat and the shape is undefined at C=0. The right edge shows that moving down increases the radius of the sphere (and approaches infinity, corresponding to a flat surface). The horizontal black line represents a physical process where the shape changes smoothly at constant curvedness. For a growing surface, the curvedness tends to decrease, hence the trajectory in (C,S)-frame is a curve pointing downwards. Figure 2b reveals the continuous evolution of the principal curvatures along the direction of the shape in going from a concave up sphere to a concave down sphere. The red curvature line bends upward to flat (cylinder) until it becomes the opposite (saddle) of the blue curvature line while the blue curvature line is left intact. From the saddle to the concave down sphere the blue line first flattens (cylinder) and then bends downwards to achieve perfect sphericity.

As discussed below, under given astigmatic flow kinematics, the geometric evolution is defined by a line A(C,S)=m in the (C,S)-plane, and two important growth modes appear (the exact definition of *m* is given in Section 2.2):

1. Constant shape evolution, where *S* remains as a constant and *C* decreases, following vertical downward lines in Figure 2a. m=0 or ±∞ at this mode;
2. Variable shape-variable curvedness evolution: here *m* is a nonzero constant and both quantities change, following a curve in Figure 1b.

### 2.2. Astigmatic Flows: Evolving Surfaces by Constant Normal Surface Velocity

The surface velocity v can the decomposed to a tangential component and a normal component U+Vk, where k is the normal unit, U is the surface tangential velocity, and *V* is the speed of normal motion. The time derivatives of the Gaussian *K* curvature, mean *H* curvature and deviatoric *D* curvature are (see Appendix B) [47,48,49]:

(5) $$\begin{array}{cc} \dot{K}= & U\cdot \nabla_{s}K+2HKV+H\nabla_{s}^{2}V-Dq:\nabla_{s}\nabla_{s}V\\ \dot{H}= & U\cdot \nabla_{s}H+\left(H^{2}+D^{2}\right)V+\frac{1}{2}\nabla_{s}^{2}V\\ \dot{D}= & U\cdot \nabla_{s}D+2HDV+\frac{1}{2}q:\nabla_{s}\nabla_{s}V \end{array}$$

For growth where U=0 and V=constant, Equation (5) reduces to

(6) $$\frac{\dot{H}}{\dot{D}}=\frac{H^{2}+D^{2}}{2HD}=\frac{1}{2}\left(\frac{H}{D}\right)+\frac{1}{2}{\left(\frac{H}{D}\right)}^{-1}$$

The integration of Equation (6) yields the following equivalent relations of pure growth under constant normal velocity:

(7) $$H^{2}-D^{2}=-mD\;\mathrm{or}\;\frac{1}{\kappa_{1}}-\frac{1}{\kappa_{2}}=\frac{2}{m}\;\mathrm{or}\;C=\frac{m\cos(\frac{S\pi }{2})}{\cos S\pi }$$

where *m* is a constant (constraint) depending on the shape, equivalent to the condition: $\dot{D}/D=\dot{K}/K$. A surface satisfying Equation (7) is known as a Weingarten surface, an important class of surfaces in geometry and physics [50,51] that arises naturally for the chosen flow kinematics in our model. Hence an astigmatic flow, in a mathematical sense, implies a process of a surface transforming from a Weingarten surface to another by decreasing the Casorati *C* curvature, while staying in the same class (*m* is an invariant). The distinguishing values of *m* found from Equation (7), for the primitive shapes are:

1. Sphere (S=±1), m→−∞;
2. Cylinder (S=±1/2), m→0;
3. Saddle (S=±0), m>0.

Following Equation (5), the variations of all the geometric properties mentioned above are summarized in Table 2.

An important observation is that for the primitive shapes, the rates of *C* and *S* are out of phase, and when one is zero the other is not zero, reflecting the decoupled nature of these geometric descriptors. We note that in this model, the sign of the shape parameter *S* is equal to the sign of the normal speed *V*, and when S=0, *V* has a discontinuity which exactly corresponds to saddle at which dC/dt=0 (see Figure 1b). This discontinuity in the speed introduces no inconsistency or weakness in the model since S=0 is the end point of astigmatic growth for any m>0.

In partial summary, in this section, we showed how astigmatic flow follows from the constant normal velocity kinematics and yields a series of evolving Weingarten surfaces, whose principal curvatures are constrained by an invariant *m*.

### 2.3. Generalized Boussinesq-Scriven Dissipation for Surfaces, Interfaces and Membranes

In this section, we characterize the key features of the rate of entropy production surface using the geometric (C,S)-frame.

The generalized curvature dissipation Δ for a Boussinesq-Scriven surface fluid is defined by the contraction between the surface viscous moment tensor $M_{s}$ and the objective Zaremba-Jaumann derivative of curvature tensor $\overset{▵}{b}$ [45,52,53]

(8) $$\Delta =M_{s}:\overset{▵}{b}=4\eta^{b}{\dot{H}}^{2}+4\eta^{tt}{\dot{D}}^{2}+4\eta^{tt}{\dot{T}}^{2}$$

where $\eta^{b}$ and $\eta^{tt}$ are the bending and torsion/twist viscosities, and $\dot{T}$ is the twist rate. For the astigmatic flow $\dot{T}=0$, and we assume that $\eta^{b}=\eta^{tt}$ for simplicity. The detailed studies related to Equation (8) can be found in [42,54,55,56]. Rewriting Equation (5) by using Equation (3) in the (C,S)-frame and scaling all the geometric parameters with the constant normal velocity as well as the bending and torsion/twist viscosities, we find the scaled time rate of change of the average and deviatoric curvatures:

(9) $${\dot{H}}_{*}=C^{2}\;\mathrm{and}\;{\dot{D}}_{*}=C^{2}\sin S\pi$$

In the following content, the parameters are all scaled unless further specified, and the ∗ is neglected for brevity. Under U=0, the rate of entropy production Δ for a Boussinesq-Scriven surface fluid or membrane is a quadratic function of mean curvature rates $\dot{H}$ and deviatoric curvature rates $\dot{D}$, i.e., $\Delta ={\dot{H}}^{2}+{\dot{D}}^{2}>0$ in the absence of twist rate $\dot{T}=0$. In the (C,S)-frame, the entropy production rate Equation (8) becomes

(10) $$\Delta =\underset{\Delta_{H}}{\underset{︸}{C^{4}}}+\underset{\Delta_{D}}{\underset{︸}{C^{4}\sin^{2}S\pi }}=C^{4}\left(1+\sin^{2}S\pi \right)$$

where $\Delta_{H}$ is the contribution brought by the changing rate of the mean curvature and $\Delta_{D}$ is the contribution from the changing rate of the deviatoric curvature. The structure of this expression follows from the fact that the deviatoric curvature measures the sphericity and that is why $\Delta_{D}$ depends on *S*. More revealing insights are found by using the Jacobian J of the transformation. J (in Equation (4)) can be decomposed into $J=\left[\begin{array}{cc} \partial_{C}D & \partial_{S}D \end{array}\right]$. Then from the total derivative $\dot{D}=\left(\partial_{C}D\right)\dot{C}+\left(\partial_{S}D\right)\dot{S}$), we find that

(11) $$\Delta ={\dot{D}}^{2}=\underset{\Delta_{C}}{\underset{︸}{{\left(\partial_{C}D\right)}^{2}{\dot{C}}^{2}}}+\underset{\Delta_{S}}{\underset{︸}{{\left(\partial_{S}D\right)}^{2}{\dot{S}}^{2}}}$$

where $\Delta_{C}$ corresponds to the changing rate of curvedness and $\Delta_{S}$ the changing rate of shape. From $\dot{S}=J^{-1}\cdot \dot{D}$, $\dot{S}$ becomes

(12) $$\dot{S}=\left[\begin{array}{c} C^{2}f\left(S\right)\\ Cg(S) \end{array}\right]=\frac{2}{\pi C}\left[\begin{array}{cc} \frac{\pi }{2}C\sin\left(\frac{S\pi }{2}\right)\; & \frac{\pi }{2}C\cos\left(\frac{S\pi }{2}\right)\\ \cos(\frac{S\pi }{2}) & -\sin(\frac{S\pi }{2}) \end{array}\right]\cdot \left[\begin{array}{c} C^{2}\\ C^{2}\sin S\pi \end{array}\right]$$

where f(S) and g(S) can be read off directly from Equation (12). Hence in the (C,S)-frame, Δ is decomposed into two explicit components of shape and curvedness:

(13) $$\Delta \left(C,S\right)=\underset{\Delta_{C}}{\underset{︸}{C^{4}{\left[\sin\left(\frac{S\pi }{2}\right)+\sin S\pi \cos\left(\frac{S\pi }{2}\right)\right]}^{2}}}+\underset{\Delta_{S}}{\underset{︸}{C^{4}{\left[\cos\left(\frac{S\pi }{2}\right)-\sin S\pi \sin\left(\frac{S\pi }{2}\right)\right]}^{2}}}$$

Equation (13), as well as f(s) and g(s), can also be derived directly from the variation of *C* and *S* (see Appendix B).

The significances of these results are:

1. Equation (13) gives a direct connection between dissipation rate and geometry;
2. Equation (12) shows explicitly under which conditions shape rates vanish.

## 3. Results and Discussion

We use the following diagram to incorporate all the results stated in Section 3.


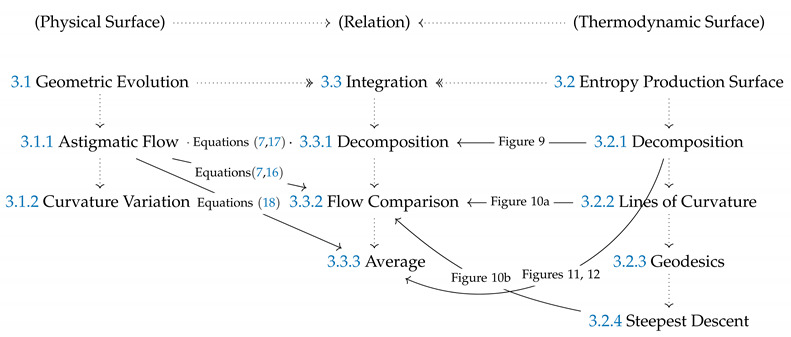


Section 3.1 presents computations, analysis and characterization of the geometric evolution of the physical surfaces under astigmatic flow and curvature variations. Section 3.2 focuses on the rate of entropy production surface and its geometric properties such as lines of curvature (Section 3.2.2), geodesics (Section 3.2.3), lines of steepest descent and level set curves (Section 3.2.4). Section 3.3 presents the complete integration of Section 3.1 and Section 3.2 and establishes the connections between geometric flows and the thermodynamic flows (Section 3.3.2), and formulates the governing scaling laws (Section 3.3.3).

### 3.1. Geometric Evolution of Surfaces, Interfaces and Membranes

#### 3.1.1. Astigmatic Flow

**Figure 3:**
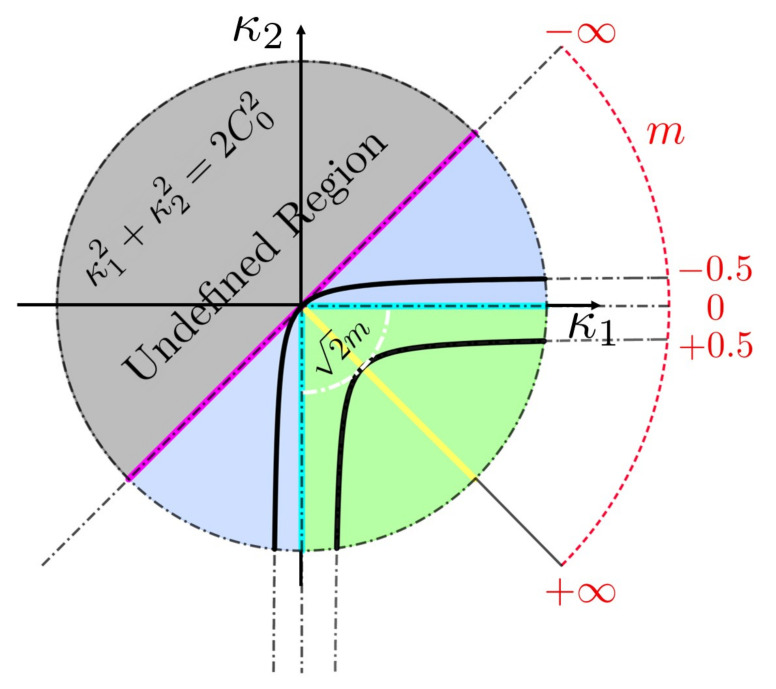
Astigmatic flow curves in the $\kappa_{1}-\kappa_{2}$ plane. The magenta line represents a sphere, the yellow line represents a saddle, and the cyan line represents a cylinder. The center is a flat surface. The blue and green regions are the transition regions similar to Figure 1. The two black curves correspond to representative astigmatic flows when m=−0.5 and +0.5. The undefined region is when $\kappa_{2}>\kappa_{1}$. The outer red dash arc shows the corresponding values of the invariant *m*; for a sphere m→−∞, for a cylinder m=0, and for saddles m>0.

The nature of astigmatic flow and the meaning of the invariant *m* is shown in Figure 3 of the maximum curvature ($\kappa_{1}$)-minimum curvature ($\kappa_{2}$) plane with representative astigmatic flow evolution curves. Figure 3 is a plot of Equation (7) with the invariant *m* as a parameter. We recall that *m* can be any real number.

The radius of a circle in this plane divided by $\sqrt{2}$ is the Casorati curvedness *C*. Radial lines in this plane corresponds to the primary shapes: yellow (saddle, m>0), cyan (cylinder, m=0), and magenta (sphere, m→−∞). During astigmatic growth, the initial state moves towards the origin, where the four lines meet, representing the flat plane. The black dot-dash circle represents a given initial curvedness $C_{0}$. If m<0, the astigmatic flow stays inside the blue region (elliptic evolution) and terminates when both principal curvatures vanish. If m>0, the evolution curve stays inside the green region (hyperbolic evolution) and ends when it reaches the intersection with the inner white arc with radius $\sqrt{2}m$. The +∞ in the outer *m* arc means the following: for any m>0 the surface geometry evolves inwards until it reaches the saddle (yellow) line (see black lines); if the value of *m* is small the end point on the saddle line is close to the origin (flat surface) while if the value of *m* is an infinitely large positive number the end point on the saddle line is infinitely far away from the center. The significance of Figure 3 is that it graphically shows the meaning of a family of Weingarten surfaces defined by the invariant *m*.

**Figure 4:**
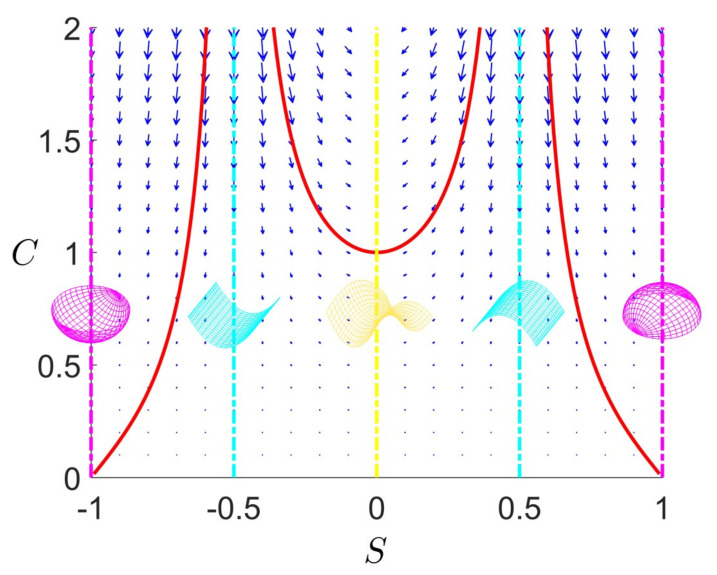
The phase flow diagram ($\dot{S},\dot{C}$) for astigmatic flow (Equation (7)). The cylindrical (S=±0.5) repulsive manifold and attractive spherical (S=±1) and saddle (S=0) manifolds are seen.

Figure 4 shows the astigmatic flow lines in the phase plane diagram, where the vector field is computed from Table 3. The figure clearly shows that the saddle line and sphere line are attractive manifolds and that the cylinder is a repelling manifold, and intensity of these effects decrease as we approach the flat plane line (C=0). The astigmatic flow exactly follows the vector field.

#### 3.1.2. Rate of Curvature Change in Evolving Surfaces under Astigmatic Flow in (*C*,*S*)-Frame

The Boussinesq-Scriven model [1] gives the entropy production in terms of rates of curvature changes. The various curvatures rates can be expressed under the (C,S)-frame, giving rise to characteristic power laws as Table 3, which is a direct result from replacing Equation (3) to Table 2.

Table 3 shows five rates of changing geometry under astigmatic flow. All these rates are proportional to *C*, $C^{2}$ or $C^{3}$. An important parameter in Table 2 and Table 3 is the velocity. As mentioned above, the sign of the velocity affects the direction of the evolution, so it takes the same sign as the shape parameter *S*. The analysis on both Table 2 and Table 3 by considering the sign of velocity we find that the governing symmetries are:

1. Odd functions such that odd(S)=−odd(−S): *H*, *S* and $\dot{H}$, $\dot{S}$;
2. Even functions such that even(S)=even(−S): *K*, *D*, *C* and $\dot{K}$, $\dot{D}$, $\dot{C}$.

Table 3 also summarizes implicitly the conditions at the end of the growth process:

1. m→−∞: Uniform spherical growth, which ends at C=0 so all the geometric rates vanish;
2. m<0: The astigmatic flow ends at C=0 and S=±1, which implies the patch will ending being a sphere. Hence all the geometric rates vanish;
3. m=0: Uniform cylinder growth, which also ends at C=0 and all geometric rates vanish;
4. m>0: The astigmatic flow ends at C=m and S=0. But the velocity has a discontinuity here. So all the geometric rates vanish too.

Since every term has a common factor (cos(Sπ/2)/cos(Sπ)), the curvatures change rapidly around a cylinder and vanish at a sphere. Sphere, cylinder and saddle are the only three possible geometries satisfying the condition that all the geometric rates vanish.

A significant experimental verification of $\dot{C}$ in Table 3 can be found in the cell growth dynamics on a biological scaffold (pure growth, no tangential velocity along the scaffold surface), which satisfies $\dot{C}∼C^{2}$ [14].

In partial summary, we have shown the scaling laws for the geometric evolution of physical surfaces and determined important end-of-growth conditions. Experimental validation of cylindrical growth shows the practical applicability of the present method.

### 3.2. Thermodynamic Surface

#### 3.2.1. Entropy Production Rate Surface and its Decomposition

To better characterize the dissipation we analyze the primitive curvature contributions to the rate of entropy production given by Equations (10), (13), and (11).

**Figure 5:**
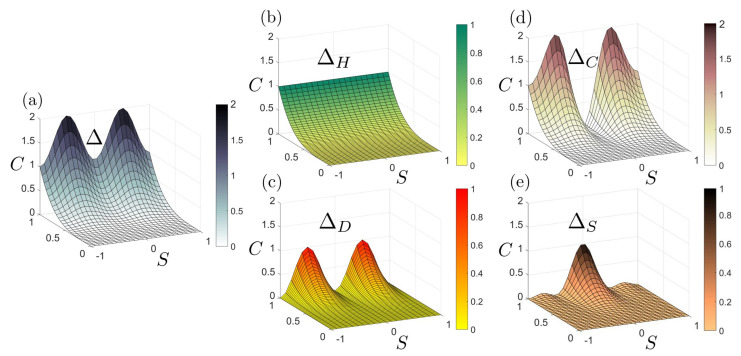
Decomposition of entropy production rate Δ into four fundamental curvature contributions. (**a**) The entropy production rate surface Δ. (**b**) The mean curvature entropy production rate $\Delta_{H}$. (**c**) The deviatoric curvature entropy production rate $\Delta_{D}$. (**d**) The curvedness entropy production rate surface $\Delta_{C}$. (**e**) The shape parameter entropy production rate $\Delta_{S}$. The figures are computed using Equations (10), (13), and (11). Comparing (**b**) and (**c**), all the shape information is contained in the deviatoric curvedness, which is why it commonly appear in the definition of the sphericity index.

The entropy production rate Δ and two decompositions are shown in Figure 5. Figure 5a is the entropy production rate surface. Figure 5b,c correspond to the mean $\Delta_{H}$ and deviatoric curvature $\Delta_{D}$ contribution, respectively. Figure 5d,e exhibit the curvedness contribution $\Delta_{C}$ and the shape contribution $\Delta_{S}$.

Figure 5a shows that the entropy production rate increases with the enhancement of curvedness for a patch with fixed shape, and that there is no entropy production rate for a flat surface (C=0). Surfaces with the same curvedness but different shape have different entropy production rate. Δ reaches the minimum for a sphere or saddle, and maximum for a cylinder. This can be explained by observing that

(14) $$\Delta ∼H^{2}D^{2}\leq \frac{1}{4}{\left(H^{2}+D^{2}\right)}^{2}=\frac{1}{4}C^{4}$$

where the “=” sign can only be taken if H=D, which corresponds to a cylinder. If we permutes *H* and *D* it does not show any effect on Equation (14), hence sphere (H=C, D=0) and saddle (H=0, D=C) should have the same entropy production rate. Δ-plot in Figure 5 is essentially a physical interpretation of the inequality of arithmetic and geometric means.

The mean curvature contribution $\Delta_{H}$ is independent of the shape parameter, while the deviatoric contribution $\Delta_{D}$ is influenced by both the curvedness and shape parameter. The deviatoric contribution has the same trend as the total entropy production rate due to the triviality of $\Delta_{H}$. Thus Figure 5b,c match with our previous discussions in connection with Equation (10).

The important decomposition of Δ into the sum of $\Delta_{C}$ and $\Delta_{S}$ is shown by Figure 5d,e. The curvedness contribution follows the ordering:

$$\Delta_{C}\left(\mathrm{cylinder}\right)>\Delta_{C}\left(\mathrm{sphere}\right)>\Delta_{C}\left(\mathrm{saddle}\right)=0$$

Notable there is no curvedness contribution for a saddle patch. The ordering for the shape contribution $\Delta_{S}$ has an additional local maximun:

$$0=\Delta_{S}\left(\mathrm{cylinder}\right)=\Delta_{S}\left(\mathrm{sphere}\right)<\Delta_{S}\left(\mathrm{dome}\right)<\Delta_{S}\left(\mathrm{saddle}\right)$$

For a sphere or a cylinder, there is no shape contribution to the entropy production rate. $\Delta_{S}$ reaches the local maximum at $\pm S^{†}$; here $S^{†}$ is the shape of a dome:

$$S^{†}=\frac{4}{\pi }\arctan\left(\sqrt{\frac{1}{5}\left(7-2\sqrt{6}\right)}\right)\approx 0.732$$

Notably, the absolute greatest shape contribution is brought by a saddle patch. We summarize our results in Table 4.

We note $\Delta_{C}$ and $\Delta_{S}$ do not simply represent the changing rate of curvedness (dC/dt) or shape (dS/dt), which are discussed in Table 2 and Table 3. In Equation (11), $\Delta_{C}$ and $\Delta_{S}$ are the changing rate multiplied by a compensation term (Jacobian). Again we see in Table 4, that the decoupled descriptors (C,S) provide independent thermodynamic information, not obtained with the (H,D)-frame.

In partial summary, we have shown that the entropy rate surface is periodic in *S* and monotonic in *C*, creating a complex dissipation landscape for variable shape evolutions (nonvanishing dS/dt) and simpler monotonic landscape for shape invariant growth (dS/dt=0). We are able to decompose the entropy rate surface into two basic contributions due to shape rate and curvedness rate changes and demonstrate that these two factors are essentially reversed, signaling a significant geometric competition.

#### 3.2.2. Lines of Curvature (LOC)

The geometry of the entropy rate surface is important to characterize the determination of how evolving surface shapes navigate the thermodynamic landscape. Here we compute the lines of curvatures of Δ(C,S) and seek to determine whether there are umbilical (defects) points corresponding to a spherical shape of Δ. These defects indicate a change in the topology of the curvature lines of Δ and we expect them to be located at particular primitive shapes (sphere, cylinder, saddle).

If every tangent vector v along curve γ is the principal direction of that point: $d\gamma /ds=v_{\kappa }/||v_{\kappa }||$, then γ is a line of curvature. Lines of curvature can be found through self-adjoint Weingarten map [51], which ensures the two families of lines of curvature form an orthogonal net on the surface. LOC can also be regarded as the intersection between the family of orthogonal surfaces according to Dupin’s theorem [44].

**Figure 6:**
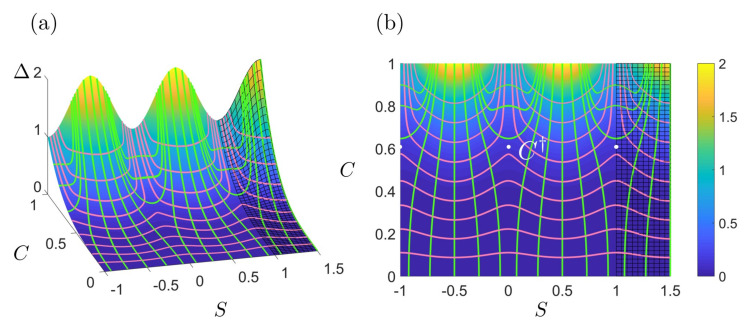
The orthogonal network of lines of curvatures in the (C,S)-frame, where *S* is extended to 1.5 for convenience, as a shaded black mesh region. (**a**) lines of curvature (LOC) network on Δ surface. (**b**) LOC network demonstrated within the (C,S)-frame. $C^{†}\approx 0.6$ is the solution of equation $16C^{8}+C^{2}-6/\pi^{2}=0$ and $\left(C^{†},\pm 1\right)$ and $\left(C^{†},0\right)$ are three umbilical points of surface Δ(C,S) (see Appendix A) located at spheres (S=±1) and saddle (S=0).

To facilitate the discussion of the LOC we extend the shape *S* coordinates to 1.5 and highlight this with a black mesh in Figure 6, where we show the entropy surface with its LOC (a) and the (C,S)-frame with the projections of the LOC (b).

Figure 6 can be generated by adopting a method we introduced in Appendix A. The orthogonal nets are composed by two families (green and pink) of LOC. The points $\left(0,C^{†}\right)$ and $\left(\pm 1,C^{†}\right)$, indicated by white dots, are special due to identical curvatures. The thermodynamic surface locally behaves like a sphere (curvature tensor b is degenerate). These points are called degenerate or umbilical points. In Figure 6, the LOC near the umbilical point $\left(0,C^{†}\right)$ display a characteristic star-like shape [44]. It can be shown that the umbilical point at $\left(C^{†},\pm 1\right)$ is the same as $\left(C^{†},0\right)$ from a *S*-continuation in Figure 6. From lower *C* to higher *C*, the pink LOC undergoes a transition from a smooth curve (straight if C→0) to two separate curves. Same for the green LOC from higher *C* to lower *C*; the critical point where the transition occurs is the umbilical point. Umbilical points are found in liquid crystal defects [57,58,59,60,61,62,63,63] and critical points in dynamical system [64]. An additional observation is that the lines of maximum curvatures (green) follow the maximal dissipation around cylindrical shapes, while the minimum curvature lines follow the saddle shape above the central umbilical point.

In partial summary, in this section based on the lines of curvature on the rate of entropy surface, we have shown a significant correspondence between the physical surface and the thermodynamic geometry:

saddleandsphericalphysicalsurfaces⟺sphericalthermodynamicsurfaces

Notably, we also found that the saddle and sphere have the same entropy production rate, although the are from different contributions ($\Delta_{S}\left(\mathrm{saddle}\right)=\Delta_{C}\left(\mathrm{sphere}\right)$, and $\Delta_{C}\left(\mathrm{saddle}\right)=\Delta_{S}\left(\mathrm{sphere}\right)$ ).

#### 3.2.3. Geodesics

Calculation of the geodesic lines [44] for the rate of entropy production surface identifies the shortest paths in the growth processes, which can then be used in conjunction with the astigmatic lines to understand dissipation rates.

A geodesic is a curve connecting two points on a surface with the minimum arc-length [44]. It should also be noticed that geodesics are not unique. It is possible that there are infinite geodesics between two points, for example, every meridian is a geodesic curve connecting the north pole and south pole of the earth surface. The geodesic equation in our surface is given by (see Appendix A):

(15) $$\frac{d^{2}S}{ds^{2}}=-\frac{1}{\sqrt{g}}\left(b:\frac{dS}{ds}⊗\frac{dS}{ds}\right)\partial_{S}\Delta$$

where *s* is the arc-length and *g* is the metric. We need two pairs of boundary conditions to solve Equation (15). Either by giving the coordinate of the starting point and the derivative (shooting method) [65], or by giving the coordinate of the starting and ending points (relaxation method) [66].

**Figure 7:**
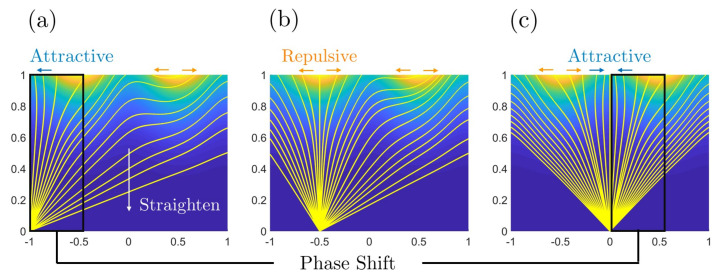
The geodesics of surface Δ projected on the (C,S)-frame. The starting points are (−1,0), (−0.5,0) and (0,0) from left to right. The color is the same as Figure 1.

Figure 7 shows the geodesics of the thermodynamic surface on the (C,S)-frame for three starting points, using the shooting method to solve Equation (15).

If we compare Figure 7a,c, we find that the region inside the black rectangle is the same. Similar behaviour can be observed in Figure 6 where the *S* is continued until 1.5. From S=−1 to −0.5 and from S=0 to 0.5, the geodesic curve implies a phase shift. This also verifies that the sphere and saddle have the same overall entropy behaviour. Another important observation is that the closer the geodesics are to C=0, the straighter geodesics are due to small curvedness.

If we move to a higher curvedness region (C=1) and regard the yellow curves as trajectories that began from the starting point. We found that when yellow curves approach C=1, they are repulsed by S=−0.5, but attracted by S=0 or S=±1. The geodesic curve hits S=−0.5 if and only if it starts at S=−0.5.

In Figure 7 the top text identifies attractive and repulsive manifolds. In particular we see that the geodesics are repelled from the cylinder (S=−1/2, in (b)) and clearly attracted for spheres (S=−1, (a)) and saddles (S=0, (c)). These results are consistent with the phase plane diagram shown in Figure 4. We note that the cylindrical manifold is repulsive and therefore a generator of saddle-like surfaces. On the other hand geodesic flow around spheres and saddles show the attractive nature of these shape manifolds.

The significance of the geodesic lines calculation includes the entropy production rate minimization if the surface evolution is along the geodesic curve in pure growth kinematics since the cylinder path corresponds to maximal entropy production rate and is a repulsive manifold. In addition, |S|=0.5 corresponds to a cylinder (K=0), which is the physical interpretation of Theorema Egregium [44].

#### 3.2.4. Lines of Steepest Descent and Level Set Curves

From a dissipation standpoint, the orthogonal family of curves of steepest descent and level set curves have a central role. Knowing in which direction the entropy rate decreases the fastest in the (C,S)-frame will help classify evolutions into distinguishing classes involving shape transitions.

**Figure 8:**
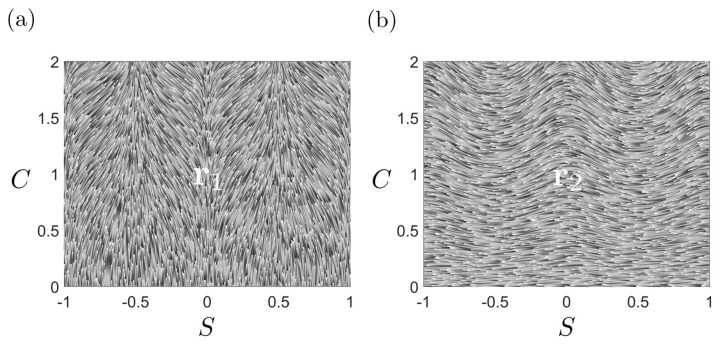
(**a**) Curves of steepest descent family $r_{1}$ obtained from Equation (16). The curves demonstrate that the sphere, cylinder and saddles are the envelopes (common tangent) to these curves. (**b**) $r_{2}$ is the other eigenvector field of tensor g and are everywhere orthogonal to $r_{1}$, and correspond to the level set curves where Δ is a constant. Figure is generated by the algorithm from [67].

As shown in the Appendix A, the curves of steepest descent are found from the eigenvector of the first fundamental form tensor g. The (C,S)-curves of steepest descent and level set curves are:

(16) $$\begin{array}{cc} r_{1}:\; & \ln\left|\frac{\tan S\pi }{\cos S\pi }\right|+\chi =\frac{\pi^{2}}{4}C\\ r_{2}:\; & 3-\cos2S\pi +\chi =\sqrt[{4}]{C} \end{array}$$

where χ is a constant. The two eigenvector fields of tensor g are the steepest descent curves $r_{1}$ (Figure 8a) and the orthogonal complement $r_{2}$ (Figure 8b); here $r_{1}$ correspond to the gradient field of the entropy production rate surface and $r_{2}$ to the level set curves.

Using Equation (16) we find that

$$\mathrm{along}\;r_{1}:\;{\left.\frac{dC}{dS}\right|}_{S=\pm 1,\pm 1/2,0}\to \infty$$

thus all primitive shapes are envelopes (tangents) of the steepest descent family of curves. The cylinder and saddle are also cusp loci since the dC/dS switches between −∞ to +∞.

On the other hand, for the *S*-periodic level set curves we find:

$$\mathrm{along}\;r_{2}:\;{\left.\frac{dC}{dS}\right|}_{S=\pm 1,\pm 1/2,0}=0$$

which is importance to characterize the formation of saddles.

In partial summary, the steepest descent curves for the rate of entropy production surface decorate the (C,S)-plane with the envelope curves that are exactly the primary shapes. The end points of all these curves are the flat plane C=0 and hence fastest decrease of the entropy production rate implies curvedness decrease and shape invariance. On the other hand, the *S*-periodic level set curves indicate that saddles form when the dissipation rate remains constant.

### 3.3. Integration of Physical Surface Geometry and Entropy Production

#### 3.3.1. Entropy Dissipation of Astigmatic Flows

The final modelling stage is to insert the constraint that controls the (C,S)-evolution, into the entropy production rate surface and find the sought after actual connections between physical surface geometry evolution and rate of entropy production by torsion and bending deformation rates.

The entropy production rate (Equations (10) and (13)) along the astigmatic flow (Equation (7)) can be uniquely written as a function of *S* or *C* only:

(17) $${\Delta |}_{A}={\left[\frac{m\cos(\frac{S\pi }{2})}{\cos S\pi }\right]}^{4}\left(1+\sin^{2}S\pi \right){\;\mathrm{or}\;\Delta |}_{A}=C^{4}\left[2-\frac{m^{2}{\left(\sqrt{8C^{2}+m^{2}}+m\right)}^{2}}{16C^{4}}\right]$$

It can be shown that ${\Delta |}_{A}$ decreases when S→0 or S→±1 from *S* cloase to ±0.5. Hence, the entropy production rate declines along the direction of surface evolution.

For coupled variable shape-variable curvedness mode we can use either Equation (17). For the uncoupled constant shape-variable curvedness mode we find:

$$\begin{array}{cc} \mathrm{Cylinders}:\; & {\Delta \left(C\right)|}_{A}=2C^{4};\;S=\pm 1/2,m=0\\ \mathrm{Spheres}:\; & {\Delta \left(C\right)|}_{A}=C^{4};\;S=\pm 1,m\to -\infty \end{array}$$

**Figure 9:**
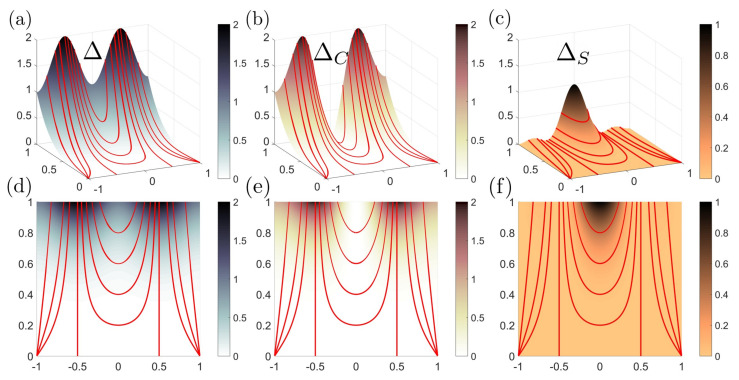
The projection of astigmatic evolution curves on the surface of entropy production rate Δ (**a**,**d**) and its two components $\Delta_{C}$ (**b**,**e**) and $\Delta_{S}$ (**c**,**f**). The highly curved astigmatic lines follow the contour of the $\Delta_{S}$ shape while the more linear astigmatic lines are found along the steepest decent of $\Delta_{C}$.

Figure 9 shows how the flow lines are embedded in the various entropy surfaces Δ, $\Delta_{C}$, $\Delta_{S}$. Since $\Delta_{C}$, $\Delta_{S}$ are out-of-phase with respect to shape, we find that astigmatic flow lines are more curved (less curved) around saddles (cylinders). The net effect is that for Δ at higher *C*, the lines splay out from the higher entropy production rate towards lower dissipative shapes.

The astigmatic flows are divided mainly by two regions in Figure 9.

1. If m<0, then 0.5<|S|<1 (between sphere and cylinder, see Figure 1), all the astigmatic flows are distributed separately in region −1<S<−0.5 and 0.5<S<1. And the astigmatic flows eventually end to S=±1, implying that the surface only evolves to a flat plane (C=0). If m→0, then astigmatic flow becomes S=±1/2. And if m→−∞, the astigmatic flow becomes S=±1.
2. If m>0, then |S|<0.5 (S≠0), all the astigmatic flows are distributed within −0.5<S<0.5. And the astigmatic flows terminate at S=0, which implies that the surface terminates to a saddle, whose curvedness is a non-zero value *m*.
3. If m=0, the astigmatic flow becomes S=±1/2. This results in a uniform growth for a surface with vanishing Gaussian curvature, and this is the Theorema Egregium. The astigmatic flow and the growth along geodesics only match in the situation where the surface is locally a cylinder. The astigmatic flows imply that sphere (most of the particle) and saddle (minimal surface) are two stable geometric exhibitions of a small patch. However, a small disturb on a cylindrical surface will result in a bifurcation to a sphere or a saddle, performing as a repelling manifold.

The termination of the evolutions leads to vanishing geometric rates (Table 2 and Table 3), which reveals that there can only be three possible final states: infinite sphere (D=0), infinite cylinder (K=0) or saddle (H=0). We note that the former two end points are flat surfaces.

In partial summary, in this section, we showed the sensitivity of the geometric evolution to the initial state, such that only spheres and cylinders grow with constant shapes. Perturbed cylinders grow either to more spherical patches or towards saddle patches. The growth terminates on either a flat surface or in a perfect saddle. Hence cylinders are a rich source of geometric diversity.

#### 3.3.2. Relationship Between Astigmatic Flow and Thermodynamic Geometric Flows

As expected astigmatic flow lines (C=C(m,S)) of evolving physical surfaces and thermodynamic geometric flows such as lines of curvature (LOC) of the entropy production surface or the phase diagram are strongly connected.

**Figure 10:**
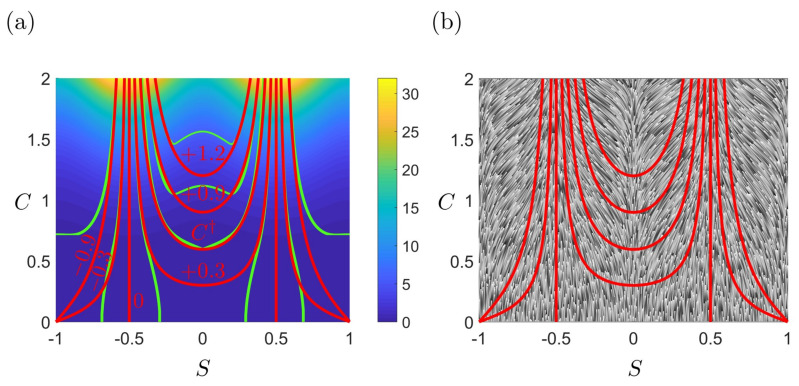
The relationship between astigmatic flows and thermodynamic geometric flows. (**a**) The red astigmatic flow (red numbers are the corresponding *m* values) and the green lines of curvature (see Figure 6) essential superpose for trajectories traversing the umbilical defect point $C^{†}$. (**b**) The red astigmatic flow and the steepest descent curve family (see $r_{1}$ in Figure 8). The set of lines are closer to each other only near cylinders.

From Figure 10a we note that in the saddle-cylinder region (−0.5<S<0.5), the astigmatic flow does not exactly follow the LOC of the entropy production rate surface, however, the minimum distance between them occurs when both the astigmatic flow and the LOC pass through the umbilic point $C^{†}$ of the entropy production rate surface. The umbilic is also the defect point from which, a large difference between the astigmatic flow and LOC sets in at lower *C*.

At high curvedness *C* in the saddle-cylinder region (−0.5<S<0.5), the astigmatic flow and the lines of curvature superpose very well. The green LOC is the maximum curvature line in Figure 6 (green). This result reveals that when the physical growth starts at a very high curvedness state, the evolution line follows the maximum curvature line of the thermodynamic surface.

A similar concept to maximum curvature is the steepest descent curve. If we project the astigmatic flow on the steepest descent curve family, it can be shown that they match more in the cylinder-sphere region (|S|>0.5) rather than in the saddle-cylinder region, as shown in Figure 10b. The connections between astigmatic flow and thermodynamic geometric flows are summarized by Table 5.

In partial summary, we established in this section that at sufficiently high initial curvedness, saddle ruts and saddles ridges (nomenclature adapted from Figure 1b) evolve essentially along the maximal lines of curvatures of the entropy production surface. The umbilic defect point in the entropy production surfaces corresponds to saddle surface and marks the departure of surface evolution lines from the entropy production curvature lines.

#### 3.3.3. Average Entropy Production Rate

In the final stage, we integrate the evolving surface geometry under bending and torsion rates with the Boussinesq-Scriven entropy production, which is a main objective of this paper. In essence we wish to establish what is the average dissipation $\bar{\Delta }$ for a astigmatic geometric change from an initial state $\left(C_{0},S_{0}\right)$ to a final state $\left(C_{f},S_{f}\right)$ and find any thermodynamic scaling laws:

$$\left(C_{0},S_{0}\right)\overset{\mathrm{Average}\;\mathrm{Entropy}\;\mathrm{Production}\;\bar{\Delta }}{\underset{\mathrm{astigmatic}\;\mathrm{flow}\;A}{\to }}\left(C_{f},S_{f}\right)\Rightarrow \bar{\Delta }=\alpha X^{n}$$

where {α,X,n} need to be found. We note that since A(C,S,m)=0, if *m* is given then the initial state is defined by either $C_{0}$ or $S_{0}$. We can evaluate the average entropy production rate Δ in terms of either the curvedness ${\bar{\Delta }}_{C}$ or the shape parameter ${\bar{\Delta }}_{S}$, defined by

(18) $${\bar{\Delta }}_{C}\left(m\right)=\frac{1}{C_{f}-C_{0}}\int_{A(m)}\Delta dC\;\mathrm{and}\;{\bar{\Delta }}_{S}\left(m\right)=\frac{1}{S_{f}-S_{0}}\int_{A(m)}\Delta dS$$

where A=A(m) represents the astigmatic flow. Equation (18) implies two important facts: (1) both ${\bar{\Delta }}_{C}$ and ${\bar{\Delta }}_{S}$ have the same units as the entropy production rate; (2) both ${\bar{\Delta }}_{C}$ and ${\bar{\Delta }}_{S}$ show singularities (caused by the vanishing denominator in Equation (18)). The singularity of ${\bar{\Delta }}_{C}$ appears if the initial curvedness and the final curvedness are identical. This exceptional case only corresponds to the saddle growth or a flat surface. However, any growth keeping the same shape exhibit a singularity in the definition of ${\bar{\Delta }}_{S}$. Hence ${\bar{\Delta }}_{S}$ is not defined for all primitive shapes.

**Figure 11:**
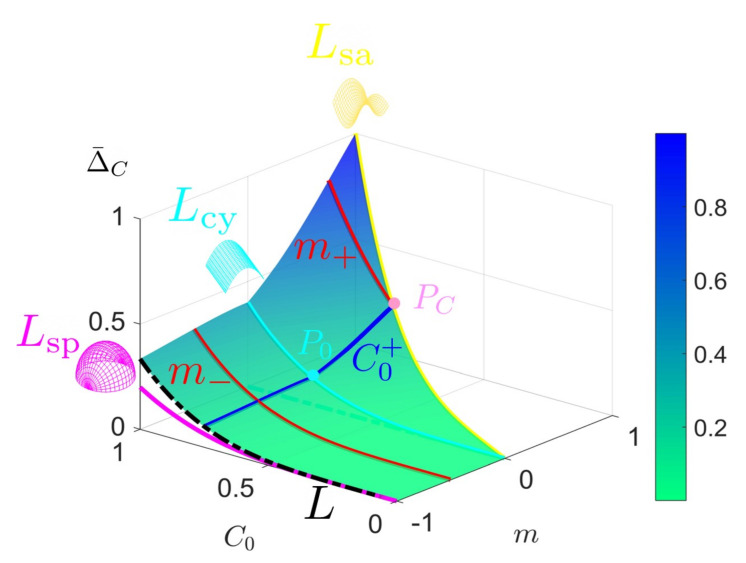
Curvedness-average entropy production rate ${\bar{\Delta }}_{C}$ as a function of the initial curvedness $C_{0}$ and invariant m. The red $m_{\pm }$ curves correspond to m=±0.5. The blue $C_{0}^{+}$ curve is generated by fixing $C_{0}=+0.75$. The magenta, cyan and yellow curves correspond to sphere growth ($L_{\mathrm{sp}}$), cylinder growth ($L_{\mathrm{cy}}$) and saddle end points ($L_{\mathrm{sa}}$), respectively.

**Figure 12:**
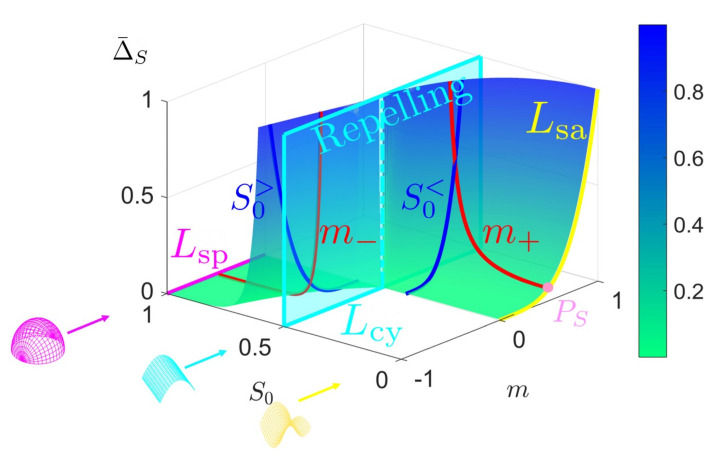
Shape-averaged entropy production rate ${\bar{\Delta }}_{S}$ as a function of the initial shape $S_{0}$ and invariant *m*. We use the same nomenclature as in Figure 11. The two separate blue lines, $S_{0}^{>}$ (here, $S_{0}=0.6$) and $S_{0}^{<}$ (here, $S_{0}=0.4$) correspond to $S_{0}>0.5$ or $S_{0}<0.5$. The cyan vertical flat surface is to emphasize the repelling nature of the cylindrical shape ($L_{\mathrm{cy}}$). $m_{\pm }$ is computed through m=±0.5.

Equation (18) can be solved analytically by introduction two special function, Sh(S) and Cur(C,m) (consult Appendix C). The entropy production is uniquely determined once the initial state ($C_{0}$ or $S_{0}$) and the astigmatic flow parameter (*m*) are given. The analysis on how these two variables influence on the average entropy production rate can be performed via Figure 11 and Figure 12, both of which provide simple scaling laws.

Figure 11 shows the curvedness-averaged entropy production rate ${\bar{\Delta }}_{C}$ as a function of the initial curvedness $C_{0}$ and astigmatic invariant *m*. The figure reveals most of the important features of ${\bar{\Delta }}_{C}$. The constant shape growth occurs when $m=C_{0}>0$ (saddle), m=0 (cylinder) and m=−∞. The line $L_{\mathrm{cy}}$ separates the surface to two regions. On the left side, the asymptotic curve $L_{\mathrm{sp}}$ (sphere: m=−∞) is moved to m=−1 for a comparison purpose. The black dash curve, i.e., the left edge of the ${\bar{\Delta }}_{C}$ surface, approaches $L_{\mathrm{sp}}$ (spherical line) as *m* decreases. On the right hand side, the surface terminates at the yellow (saddle) line $L_{\mathrm{sa}}$ since *m* is not allowed to exceed $C_{0}$. This process can be explained through the specific $C_{0}^{+}$ line (blue). ${\bar{\Delta }}_{C}$ increases along the positive direction of *m*, and terminates when reaching $L_{\mathrm{sa}}$ (point $P_{C}$). However, $C_{0}^{+}$ is continuous but not smooth. $C_{0}^{+}$ changes rapidly at the point $P_{0}$, whose physical correspondence is a cylinder. From our previous discussion, one can never cross the repelling manifold and smoothly obtain a hyperbolic surface from an elliptic surface, or vice versa. This fact is now verified by the broken smoothness of the average thermodynamic property.

If we seek to study ${\bar{\Delta }}_{C}$ with fixed *m*, we obtain the two red curves $m_{\pm }$. A surface evolves to a sphere by following $m_{-}$, yielding vanishing ${\bar{\Delta }}_{C}$. On the other hand, the evolution terminates in a saddle by following $m_{+}$, corresponding to the pink point $P_{C}$. The special lines in Figure 11 satisfy thermodynamic scaling laws of the form: ${\bar{\Delta }}_{C}=\alpha C_{o}^{n}$, where the amplitude α and exponent *n* are shown in Table 6. As expected for the primitive shapes the amplitude α is a function of the invariant *m* and *n* is a universal constant equal to 4. The amplitude increases with *m* and the *C*-averaged entropy production also increases with *m*. The latter reflects the fact that starting with the same $C_{0}$, cylinders and spheres quickly evolve into flat surfaces with C=0, while saddle-ruts evolve into saddles and sample higher entropy production rates because *C* remains relatively large.

The shape-averaged entropy production ${\bar{\Delta }}_{S}$ reveals quantitatively the average thermodynamic property by emphasizing the change of the shape. ${\bar{\Delta }}_{S}$ is an invariant with respect to the direction of the evolution, as well as the sign of the shape parameter. In other words, the mirror image of a surface, where the mirror is normal to the velocity of a surface evolution, shares the same thermodynamic behavior. For simplicity, we only solve ${\bar{\Delta }}_{S}$ on the region with positive shape parameter (S>0).

The shape average ${\bar{\Delta }}_{S}$ exhibit 3 singularities in Figure 12 in accordance with constant shape evolution: $S=S_{0}=\mathrm{constant}$. Hence, all the three lines $L_{\mathrm{sp}}$, $L_{\mathrm{cy}}$ and $L_{\mathrm{sa}}$ are not defined. The repelling nature of the cylinder ($S_{0}=0.5$) separates the surface into two regions, similar to the $L_{\mathrm{cy}}$ line in Figure 11.

By fixing $S_{0}$, the surface contracts to a line in the ${\bar{\Delta }}_{S}-m$ plot, however, the repelling cylindrical space introduces a singularity, and this can be clearly seen in that $S_{0}^{>}$ and $S_{0}^{<}$ are not continuous in Figure 12.

The repelling nature is more obvious by fixing *m*. Then the ${\bar{\Delta }}_{S}-S_{0}$ plot becomes similar to $\Delta -S_{0}$ plot. The $m_{-}$ line ends as a sphere and $m_{+}$ line ends as a saddle, at a point $P_{S}$.

A significant technical difference between the introduced functions $\mathrm{Sh}(S_{0})$ and $\mathrm{Cur}(m,C_{0})$ (see Appendix C) is that *m* and $C_{0}$ are commingled in Cur. The scaling law for shape-averaged entropy production rate ${\bar{\Delta }}_{S}$ is more general and is summarized to Table 7.

Fourth order curves $\alpha m^{4}$ are always seen in Figure 12, where α is only a function related to the initial shape $S_{0}$ only.

The greatest practical difference between Figure 11 and Figure 12 is that the shape-averaged entropy production rate ${\bar{\Delta }}_{S}$ has a simpler analytic form but with the cost of introducing singularities such that one cannot discuss constant shape evolutions. The curvedness average ${\bar{\Delta }}_{C}$, on the other hand, gives a more complete image involving cylindrical and spherical growth. The generality of ${\bar{\Delta }}_{C}$ credits to the fact that an evolution tends to decrease the curvedness and in the cost of changing the shape rather than maintaining the curvedness. ${\bar{\Delta }}_{C}$ and ${\bar{\Delta }}_{S}$ also share similarities such as exhibiting the repelling nature of the cylinder.

In summary, by integrating surface geometry evolution and dissipation by bending and torsion rates we found important scaling laws for average entropy production that follow quartic exponents in terms of either Cassorati (*C*) curvature or the Weingarten invariant *m*.

## 4. Conclusions

In this paper, we proposed, formulated, developed, implemented and partially validated a novel method to investigate the rate of entropy production generated under constant normal growth velocity (astigmatic flow) in deforming surfaces, interfaces, and membranes described by the Boussinesq-Scriven fluid model. The model describes surface dissipation by bending and torsion rates which are described directly by natural geometric coordinates: the Casorati curvedness and the shape parameter. This direct link connects evolving physical geometries to the rate of entropy productions and allows us to predict the role of shape and curvedness changes on dissipation rates and answer questions such as which shapes are associated with lower or higher rates entropy production.

The main characteristics of the thermodynamic rate of entropy production surface are that it decreases with decreasing curvedness but is periodic with shape changes. The cylinder is associated with the highest rates while the sphere and saddles with lowest rates. The combination of monotonicity and periodicity is reflected in the lines of curvature, geodesic lines and steepest descent curves. Umbilic defect points in the lines of curvature naturally divide the thermodynamics into high curvedness and low curvedness regions. Importantly the envelope lines of the steepest descent curves are the primary sphere, cylinder and saddle shapes. Accordingly, an efficient entropy rate decrease involves both lowering the curvedness (flattening the surface) and changing shapes from cylinders to spheres or saddles.

A growth only with constant normal velocity results in an astigmatic flow, where the principal physical surface curvatures are related by a constraint. This constraint evolution results in a particular path on the curvedness-shape space. Between spheres and cylinders, we find flow lines with weaker shape changes but between cylinders and saddle there are larger shape changes and terminal curvedness; here all growth terminates on saddles.

**Figure 13:**
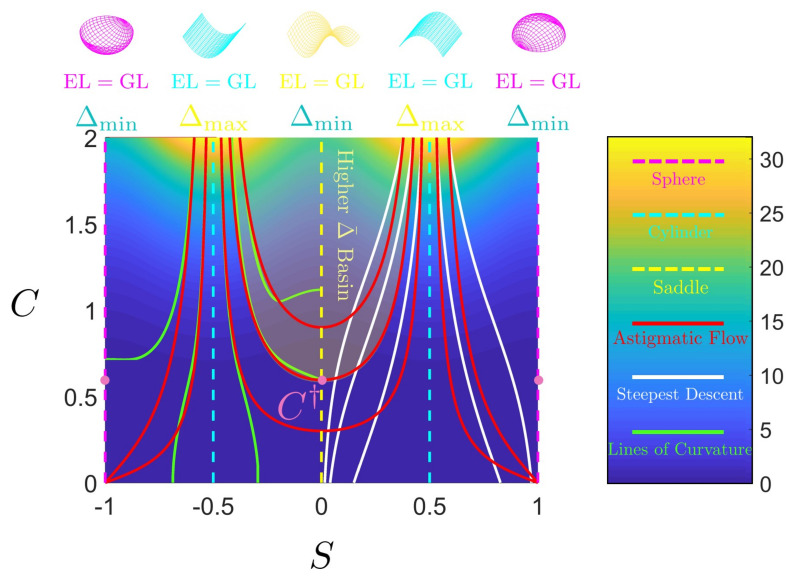
A schematic summary of the main results of this paper. From top to bottom: The first line represents the physical shape of a surface given a shape parameter. The second line shows the envelope lines (EL) and geodesic lines (GL) that only overlap along with the primitive shapes: sphere (magenta), cylinder (cyan) and saddle (yellow). The third line reveals the fact that if the curvedness is a constant, then the entropy production rate reaches minima at saddle and sphere, and maximum at the cylinder. $C^{†}$ is the umbilic point of the Δ surface (locally sphere, so we use magenta colour), above which is the higher average entropy production rate basin. The umbilic is also the point where lines of curvature start to diverge, with minimum distance between the astigmatic flow and themselves. Both the thermodynamic surface and the physical surface show periodic and symmetric behaviours in this integrated figure.

By integrating thermodynamic geometry and physical surface geometry we find that close to cylindrical growth the evolution is close steepest descent. The average entropy production rate follows scaling laws in terms of curvedness and in terms of the Weingarten invariant. Figure 13 summarizes these findings.

Taken together, the connection between viscous dissipation and morphogenesis was established for constrained bending and torsion rates of surfaces, interfaces and membranes. The cylindrical shape was singled out for high local dissipation rates, envelop for steepest descent of entropy rates, and generator of the saddle and sphere-like shapes. The high curvature saddle-like shapes are associated higher average dissipation.

## Figures and Tables

**Table 1:** The summary of four dimensional curvature concepts.

Curvatures	Symbols	Significance	Relations	Tensor Contractions	Principal Curvatures
Mean	*H*	Arithmetic Mean	$\sqrt{C^{2}-D^{2}}$	$\epsilon_{s}:\left(I_{s}\cdot \epsilon_{s}\cdot b\right)/2$	$(\kappa_{1}+\kappa_{2})/2$
Gaussian	*K*	Intrinsic Curvature	$H^{2}-D^{2}$	$\epsilon_{s}:\left(b\cdot \epsilon_{s}\cdot b\right)/2$	$\kappa_{1}\kappa_{2}$
Deviatoric	*D*	Sphericity Deviation	$\sqrt{H^{2}-K}$	$\sqrt{I_{s}:\left(f\cdot I_{s}\cdot f\right)/2}$	$(\kappa_{1}-\kappa_{2})/2$
Casorati	*C*	Planarity Deviation	$\sqrt{H^{2}+D^{2}}$	$\sqrt{I_{s}:\left(b\cdot I_{s}\cdot b\right)/2}$	$\sqrt{(\kappa_{1}^{2}+\kappa_{2}^{2})/2}$

**Table 2:** The summary of the rate of curvature change of pure growth. κ is the positive curvature of sphere, cylinder or saddle patch.

Parameter Changing Rate	Symbols	Pure Growth Dynamics	Sphere	Cylinder	Saddle
Mean	$\dot{H}$	$VC^{2}$	$V\kappa^{2}$	$\frac{1}{2}V\kappa^{2}$	$V\kappa^{2}$
Gaussian	$\dot{K}$	2VHK	$2V\kappa^{3}$	0	0
Deviatoric	$\dot{D}$	2VHD	0	$\frac{1}{2}V\kappa^{2}$	0
Casorati	$\dot{C}$	$\frac{V}{C}H\left(H^{2}+3D^{2}\right)$	$V\kappa^{2}$	$\frac{\sqrt{2}}{2}V\kappa^{2}$	0
Shape Parameter	$\dot{S}$	$-\frac{2V}{\pi C^{2}}DK$	0	0	$\frac{2}{\pi }V\kappa$

**Table 3:** Geometric Rates Power Laws.

Parameter Changing Rate	Symbols	Pure Growth Dynamics	Sphere	Cylinder	Saddle
Mean	$\dot{H}$	$VC^{2}$	$VC^{2}$	$VC^{2}$	$VC^{2}$
Gaussian	$\dot{K}$	$-2VC^{3}\sin\left(\frac{S\pi }{2}\right)\cos S\pi$	$\pm 2VC^{3}$	0	0
Deviatoric	$\dot{D}$	$VC^{2}\sin S\pi$	0	$\pm VC^{2}$	0
Casorati	$\dot{C}$	$VC^{2}\sin\left(\frac{S\pi }{2}\right)\left(\cos S\pi +2\right)$	$\pm VC^{2}$	$\pm \sqrt{2}VC^{2}$	0
Shape Parameter	$\dot{S}$	$\frac{2}{\pi }VC\cos\left(\frac{S\pi }{2}\right)\cos S\pi$	0	0	$\frac{2}{\pi }VC$

**Table 4:** The summary of shape and curvedness contribution, where *S*^†^ ≈ 0.732.

	Shape	Δ	$\Delta_{H}$	$\Delta_{D}$	$\Delta_{C}$	$\Delta_{S}$
S=0	Saddle	min	invariant	min	min (0)	max
S=±0.5	Cylinder	max	invariant	max	max	min (0)
S=±S ^†^	Ellipsoid (Dome)	/	invariant	/	/	local max
S=±1	Sphere	min	invariant	min	local min	min (0)

**Table 5:** Key Relations between thermodynamic geometry and physical surface geometry, as well as the connections between the geometric flows and astigmatic flows for primitive shapes. Here, ${\hat{\delta }}_{C}$ is the unit vector along the *C*-direction and ${\hat{\delta }}_{S}$ is the unit vector along the *S*-direction.

Primitive Physical Surface	Sphere	Cylinder	Saddle (above $C^{†}$)	Saddle (below $C^{†}$)
Principal Curvature (min, max) (Figure 6)	$\left({\hat{\delta }}_{C},{\hat{\delta }}_{S}\right)$	$\left({\hat{\delta }}_{C},{\hat{\delta }}_{S}\right)$	$\left({\hat{\delta }}_{C},{\hat{\delta }}_{S}\right)$	$\left({\hat{\delta }}_{S},{\hat{\delta }}_{C}\right)$
Geodesic Line Orientation G (Figure 7)	${\hat{\delta }}_{C}$	${\hat{\delta }}_{C}$	${\hat{\delta }}_{C}$	${\hat{\delta }}_{C}$
Steepest Descent Curves’ Envelope D (Figure 8)	${\hat{\delta }}_{C}$	${\hat{\delta }}_{C}$	${\hat{\delta }}_{C}$	${\hat{\delta }}_{C}$
Astigamtic Flow Orientation A (Figure 4)	${\hat{\delta }}_{C}$	${\hat{\delta }}_{C}$	${\hat{\delta }}_{S}$	${\hat{\delta }}_{S}$
Key Relations	(G‖D‖A‖min) ⊥max	(G‖D‖A‖min) ⊥max	(G‖D‖min) ⊥(A‖max)	(G‖D‖max) ⊥(A‖min)

**Table 6:** Summary of thermodynamic scaling laws (${\bar{\Delta }}_{C}=\alpha C_{0}^{n}$) for *C*-averaged entropy production.

Physical Shape	Sphere	Cylinder	Saddle
Special Surface Lines (Figure 11)	$L_{\mathrm{sp}}$	$L_{\mathrm{cy}}$	$L_{\mathrm{sa}}$
Invariant Values	m→−∞	m=0	$m=C_{0}$
*C*-averaged Entropy Production Scaling Law	$\frac{1}{5}C_{0}^{4}$	$\frac{2}{5}C_{0}^{4}$	$m^{4}$ or $C_{0}^{4}$

**Table 7:** Summary of thermodynamic scaling laws (${\bar{\Delta }}_{S}=\alpha m^{n}$) for shape-averaged entropy production.

Physical Shape	Sphere	Elliptic	Cylinder	Hyperbolic	Saddle
Special Surface Lines (Figure 12)	$L_{\mathrm{sp}}$	$S_{0}^{>},m_{-}$	$L_{\mathrm{cy}}$	$S_{0}^{<},m_{+}$	$L_{\mathrm{sa}}$
Invariant Values	m→−∞	m<0	m=0	m>0	$m=C_{0}$
*S*-averaged Entropy Production Scaling Law	Undefined (→0)	$\left(\frac{\mathrm{Sh}(S_{0})}{1-S_{0}}\right)m^{4}$	Undefined (→∞)	$-\left(\frac{\mathrm{Sh}(S_{0})}{S_{0}}\right)m^{4}$	Undefined ($\to m^{4}$)

**Figure A1:**
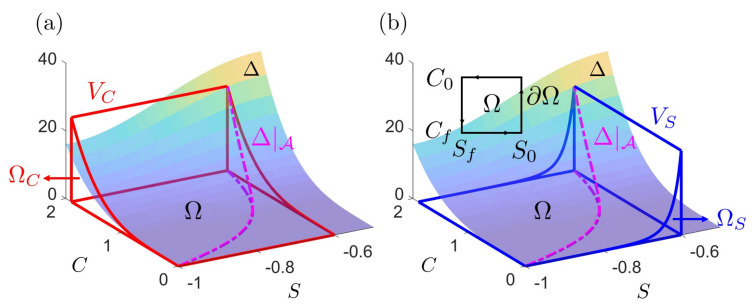
A schematic explaining the geometric meaning of the two entropy production rates. (**a**) The invariant *m* determines (here m<0) an astigmatic flow ${\Delta |}_{A}$. (**b**) blue triangular prism with one curved surface where the curve is obtained from projecting ${\Delta |}_{A}$ onto (S,Δ)-plane.

## Appendix A. Tensorial Differential Geometry

### Appendix A.1. Tensor Basis

This appendix provides background in the tensorial formulation of differential geometry from Equation (1) to Equation (4).

The 2×2 tensor bases are surface identity tensor $(I_{s}$, surface deviatoric tensor q, off-diagonal tensor $q_{1}$, and surface alternator tensor $\epsilon_{s})$. Any 2×2 tensor Z can be decomposed by using the tensor basis as [47,48]

(A1) $$Z=\underset{\mathrm{trace}}{\underset{︸}{\frac{1}{2}\left(Z:I_{s}\right)I_{s}}}+\underset{\mathrm{diagonal traceless}}{\underset{︸}{\frac{1}{2}\left(Z:q\right)q}}\;+\underset{\mathrm{anti}-\mathrm{symmetric}}{\underset{︸}{\frac{1}{2}\left(Z:\epsilon_{s}\right)\epsilon_{s}}}+\underset{\mathrm{symmetric off-diagonal}}{\underset{︸}{\frac{1}{2}\left(Z:q_{1}\right)q_{1}}}$$

with the following identities:

(A2) $$\begin{array}{c} I_{s}:I_{s}=q:q=\epsilon_{s}:\epsilon_{s}=q_{1}:q_{1}=2\\ I_{s}:q=I_{s}:\epsilon_{s}=I_{s}:q_{1}=q:\epsilon_{s}=q:q_{1}=\epsilon_{s}:q_{1}=0\\ q\cdot q_{1}=\epsilon_{s}\;\mathrm{and}\;q\cdot \epsilon_{s}=q_{1}\;\mathrm{and}\;q_{1}\cdot \epsilon_{s}=q \end{array}$$

and the following components expression

(A3) $$I_{s}=\left[\begin{array}{cc} 1 & 0\\ 0 & 1 \end{array}\right],\;q=\left[\begin{array}{cc} 1 & 0\\ 0 & -1 \end{array}\right],\;q_{1}=\left[\begin{array}{cc} 0 & 1\\ 1 & 0 \end{array}\right]\;\mathrm{and}\;\epsilon_{s}=q^{-1}\cdot q_{1}=\left[\begin{array}{cc} 0 & 1\\ -1 & 0 \end{array}\right]$$

The coefficients of decomposition of curvature tensor (no $\epsilon_{s}$ and $q_{1}$ contribution) define the mean and deviatoric curvatures

(A4) $$b=HI_{s}+Dq$$

where *H* is the mean curvature and *D* is the deviatoric tensor. *D* measures the non-sphericity index (since D=0 for a sphere). Define tensor $f=HI_{s}-b=-Dq$, then the mean curvature *H*, the Gaussian curvature *K*, squared deviatoric curvature $D^{2}$, and Casorati curvature *C* are simply the tensor contraction:

(A5) $$\begin{array}{c} H=\frac{1}{2}\epsilon_{s}:\left(I_{s}\cdot \epsilon_{s}\cdot b\right)=\frac{1}{2}\epsilon_{s}:\left(H\epsilon_{s}\cdot I_{s}+D\epsilon_{s}\cdot q\right)=\frac{1}{2}\epsilon_{s}:\left(H\epsilon_{s}-Dq_{1}\right)\\ K=\frac{1}{2}\epsilon_{s}:\left(b\cdot \epsilon_{s}\cdot b\right)=\frac{1}{2}\epsilon_{s}:\left[\left(HI_{s}+Dq\right)\cdot \epsilon_{s}\cdot \left(HI_{s}+Dq\right)\right]=H^{2}-D^{2}\\ D^{2}=\frac{1}{2}I_{s}:\left(f\cdot I_{s}\cdot f\right)=\frac{1}{2}I_{s}:\left(-Dq\cdot I_{s}\cdot -Dq\right)=\frac{1}{2}I_{s}:\left(Dq\cdot Dq\right)\\ C^{2}=\frac{1}{2}I_{s}:\left(b\cdot I_{s}\cdot b\right)=\frac{1}{2}I_{s}:\left[\left(HI_{s}+Dq\right)\cdot I_{s}\cdot \left(HI_{s}+Dq\right)\right]=H^{2}+D^{2} \end{array}$$

### Appendix A.2. Definition through Fundamental Form

This appendix presents the details of the entropy production surface appearing in Section 3.2.

The position vector on the entropy surface is r=r(C,S,Δ). We adapt Einstein notation and denote $x^{1}=C$ and $x^{2}=S$ in the following text. The surface is parametrized by $\Delta =C^{4}\left[1+\sin^{2}S\pi \right]$ with the derivatives

(A6) $$\left\{\begin{array}{c} \partial_{1}\Delta =4C^{3}\left(1+\sin^{2}S\pi \right)\\ \partial_{2}\Delta =\pi C^{4}\sin2S\pi \end{array}\right.\;\mathrm{and}\;\left\{\begin{array}{c} \partial_{11}\Delta =12C^{2}\left(1+\sin^{2}S\pi \right)\\ \partial_{12}\Delta =\partial_{21}\Delta =4\pi C^{3}\sin2S\pi \\ \partial_{22}\Delta =2\pi^{2}C^{4}\cos2S\pi \end{array}\right.$$

The tangent vectors are

(A7) $$e_{1}=\partial_{1}r={\left[\begin{array}{ccc} 1 & 0 & \partial_{1}\Delta \end{array}\right]}^{⊺}\;\mathrm{and}\;e_{2}=\partial_{2}r={\left[\begin{array}{ccc} 0 & 1 & \partial_{2}\Delta \end{array}\right]}^{⊺}$$

The metric tensor g, also known as the first fundamental form is

(A8) $$g=\left[\begin{array}{cc} 1+{\left(\partial_{1}\Delta \right)}^{2} & \partial_{1}\Delta \partial_{2}\Delta \\ \partial_{1}\Delta \partial_{2}\Delta & 1+{\left(\partial_{2}\Delta \right)}^{2} \end{array}\right]=\left[\begin{array}{cc} E & F\\ F & G \end{array}\right]$$

and the determinant is

(A9) $$g:=\det\left(g\right)=1+{\left(\partial_{1}\Delta \right)}^{2}+{\left(\partial_{2}\Delta \right)}^{2}=4C^{6}{\left(\cos2S\pi -3\right)}^{2}+\pi^{2}C^{8}\sin^{4}2S\pi +1>1$$

indicating that g is invertible with the inverse

(A10) $$g^{-1}=\frac{1}{g}\left[\begin{array}{cc} 1+{\left(\partial_{2}\Delta \right)}^{2} & -\partial_{1}\Delta \partial_{2}\Delta \\ -\partial_{1}\Delta \partial_{2}\Delta & 1+{\left(\partial_{1}\Delta \right)}^{2} \end{array}\right]$$

The first fundamental form induces arc-length d*s* by

(A11) $$ds^{2}=g:dxdx$$

The upward normal vector n becomes

(A12) $$n=\frac{e_{1}\times e_{2}}{\sqrt{g}}=\frac{1}{\sqrt{1+{\left(\partial_{1}\Delta \right)}^{2}+{\left(\partial_{2}\Delta \right)}^{2}}}{\left[\begin{array}{ccc} -\partial_{1}\Delta & -\partial_{2}\Delta & 1 \end{array}\right]}^{⊺}$$

The second fundamental form b is

(A13) $$b{}_{ij}=\partial_{j}e_{i}\cdot n=\partial_{j}\partial_{i}r\cdot n=\frac{1}{\sqrt{1+{\left(\partial_{1}\Delta \right)}^{2}+{\left(\partial_{2}\Delta \right)}^{2}}}\left[\begin{array}{cc} \partial_{11}\Delta & \partial_{12}\Delta \\ \partial_{21}\Delta & \partial_{22}\Delta \end{array}\right]=\left[\begin{array}{cc} L & M\\ M & N \end{array}\right]$$

whose corresponding mixed term becomes

(A14) $$b{}_{i}^{j}=b{}_{ik}g{}^{kj}=g^{-3/2}\left[\begin{array}{cc} \partial_{11}\Delta \left[1+{\left(\partial_{2}\Delta \right)}^{2}\right]-\partial_{12}\Delta \partial_{1}\Delta \partial_{2}\Delta \; & \partial_{12}\Delta \left(1+{\left(\partial_{1}\Delta \right)}^{2}\right)-\partial_{11}\Delta \partial_{1}\Delta \partial_{2}\Delta \\ \partial_{12}\Delta \left(1+{\left(\partial_{2}\Delta \right)}^{2}\right)-\partial_{22}\Delta \partial_{1}\Delta \partial_{2}\Delta \; & \partial_{22}\Delta \left(1+{\left(\partial_{1}\Delta \right)}^{2}\right)-\partial_{12}\Delta \partial_{1}\Delta \partial_{2}\Delta \end{array}\right]$$

Through mixed form $b_{i}^{j}$, the mean and the Gaussian curvatures are

(A15) $$\begin{array}{c} H_{\Delta }=\frac{1}{2}\mathrm{tr}\left(b_{i}^{j}\right)=\frac{LG-2MF+NE}{2(EG-F^{2})}=\frac{\partial_{11}\Delta \left[1+{\left(\partial_{2}\Delta \right)}^{2}\right]+\partial_{22}\Delta \left[1+{\left(\partial_{1}\Delta \right)}^{2}\right]-2\partial_{12}\Delta \partial_{1}\Delta \partial_{2}\Delta }{2{\left[1+{\left(\partial_{1}\Delta \right)}^{2}+{\left(\partial_{2}\Delta \right)}^{2}\right]}^{3/2}}\\ K_{\Delta }=\det\left(b_{i}^{j}\right)=\frac{\det(b)}{\det(g)}=\frac{LN-M^{2}}{EG-F^{2}}=\frac{\partial_{11}\Delta \partial_{22}\Delta -{\left(\partial_{12}\Delta \right)}^{2}}{{\left[1+{\left(\partial_{1}\Delta \right)}^{2}+{\left(\partial_{2}\Delta \right)}^{2}\right]}^{2}} \end{array}$$

Let $C_{\Delta }$ and $S_{\Delta }$ be the Casorati and deviatoric curvature of entropy surface, whose deviatoric is curvature $D_{\Delta }$, then

(A16) $$D_{\Delta }=\sqrt{H_{\Delta }^{2}-K_{\Delta }},\;C_{\Delta }=\sqrt{2H_{\Delta }^{2}-K_{\Delta }}\;\mathrm{and}\;S_{\Delta }=\frac{2}{\pi }\arctan\left(\frac{H_{\Delta }}{\sqrt{H_{\Delta }^{2}-K_{\Delta }}}\right)$$

If $D_{\Delta }=0$ at point *p*, then *p* is the umbilical point, where $S_{\Delta }$ is not defined (S→±1). Especially, we consider the following two conditions that always give $D_{\Delta }=0$:

(1) If C=0, then $g=I_{s}$, b=0 so $D_{\Delta }=C_{\Delta }=0$.

(2) If S=0 or ±1, then ${g|}_{S=0,\pm 1}=\mathrm{diag}\left[1+16C^{6},1\right]$ and ${b|}_{S=0,\pm 1}={\left(1+16C^{6}\right)}^{-1/2}\mathrm{diag}\left[12C^{2},2\pi^{2}C^{4}\right]$. The mixed form $g^{-1}{b|}_{S=0,\pm 1}={\left(1+16C^{6}\right)}^{-3/2}\mathrm{diag}\left[12C^{2},2\pi^{2}C^{4}\left(1+16C^{6}\right)\right]$. If $D_{\Delta }=0$ then $C=C^{†}$ where $C^{†}$ is the solution of equation $16C^{8}+C^{2}-\frac{6}{\pi^{2}}=0$. $\left(C^{†},\pm 1\right)$ and $\left(C^{†},0\right)$ are three umbilical points of surface Δ(C,S).

We note that if S=±1/2, it can be shown that there are no umbilical points of surface Δ(C,S).

### Appendix A.3. Lines of Curvature

This appendix formulates the algorithm to compute lines of curvature shown in Section 3.2.2.

It can be shown that the problem of lines of curvature is actually just solving the following equations [66]

(A17a) $$\begin{array}{c} \frac{dC}{ds}=\eta \left(M-\kappa F\right)\;\mathrm{and}\;\frac{dS}{ds}=-\eta \left(L-\kappa E\right) \end{array}$$

(A17b) $$\begin{array}{c} \frac{dC}{ds}=\mu \left(N-\kappa G\right)\;\mathrm{and}\;\frac{dS}{ds}=-\mu \left(M-\kappa F\right) \end{array}$$

where the non-zero factors η and μ are determined by

(A18a) $$\begin{array}{c} \eta =\frac{\pm 1}{\sqrt{E{\left(M-\kappa F\right)}^{2}-2F\left(M-\kappa F\right)\left(L-\kappa E\right)+G{\left(L-\kappa E\right)}^{2}}} \end{array}$$

(A18b) $$\begin{array}{c} \mu =\frac{\pm 1}{\sqrt{E{\left(N-\kappa G\right)}^{2}-2F\left(M-\kappa F\right)\left(N-\kappa G\right)+G{\left(M-\kappa F\right)}^{2}}} \end{array}$$

The ± sign in Equations (A18a) and (A18b) just represents the proceeding direction of solution. We can solve either differential Equation (A17a) or Equation (A17b). The following criteria is used by considering the common factor M−κF: If |L−κE|≥|N−κG|, we solve Equation (A17a), otherwise we solve Equation (A17b).

When we integrate the equations, S might change sign, therefore we have to adjust ± in Equations (A18a) and (A18b). This situation can be intuitively explained by the following diagram.

The next S should have a small angle with the previous time step ${S|}_{p}$. If the angle between +S and ${+S|}_{p}$ is larger than π/2, then red vector is shorter than the blue one, yielding

(A19) $$\left|-{\left.\left(\frac{dx^{i}}{ds}\frac{\partial r}{\partial x^{i}}\right)\right|}_{p}-\left(\frac{dx^{i}}{ds}\frac{\partial r}{\partial x^{i}}\right)\right|<\left|{\left.\left(\frac{dx^{i}}{ds}\frac{\partial r}{\partial x^{i}}\right)\right|}_{p}-\left(\frac{dx^{i}}{ds}\frac{\partial r}{\partial x^{i}}\right)\right|$$

### Appendix A.4. Geodesics

In this appendix we derive Equation (15) in Section 3.2.3.

A curve on the entropy surface is parametrized by r=r(s)=r(C(s),S(s),Δ(s)) where *s* is the arc-length. Hence

(A20) $$ds=\sqrt{dr\cdot dr}=\sqrt{r_{i}\frac{dx^{i}}{ds}ds\cdot r_{j}\frac{dx^{j}}{ds}ds}$$

The total length becomes

(A21) $$\mathrm{Length}=\int_{s_{0}}^{s_{1}}\sqrt{g_{ij}\frac{dx^{i}}{ds}\frac{dx^{j}}{ds}}s$$

Denote $x^{\prime }$ as length derivative. Taking the variance of total length such that

(A22) $$\delta \mathrm{Length}=\int_{s_{0}}^{s_{1}}\frac{1}{2}\underset{=1}{\underset{︸}{{\left(g_{ij}x^{\prime i}x^{\prime j}\right)}^{-1/2}}}\delta \left(g_{ij}x^{\prime i}x^{\prime j}\right)ds$$

Evaluate the following variation

(A23) $$\begin{array}{cc} \delta \left(g_{ij}x^{\prime i}x^{\prime j}\right)ds= & \delta g_{ij}x^{\prime i}x^{\prime j}ds+g_{ij}\delta x^{\prime i}x^{\prime j}ds+g_{ij}x^{\prime i}\delta x^{\prime j}ds\\ = & \frac{\delta g_{ij}}{\delta x^{k}}\delta x^{k}x^{\prime i}x^{\prime j}ds+g_{ij}d\delta x^{i}x^{\prime j}+g_{ij}x^{\prime i}d\delta x^{j}\\ = & \frac{\delta g_{ij}}{\delta x^{k}}\delta x^{k}x^{\prime i}x^{\prime j}ds+d\left(g_{ij}\delta x^{i}x^{\prime j}\right)-d\left(g_{ij}x^{\prime j}\right)\delta x^{i}\\ \; & +d(g_{ij}x^{\prime i}\delta x^{j})-d\left(g_{ij}x^{\prime i}\right)\delta x^{j} \end{array}$$

The variation of the length can be re-written as

(A24) $$\begin{array}{cc} \delta \mathrm{Length}= & \int_{s_{0}}^{s_{1}}\frac{1}{2}\delta \left(g_{ij}x^{\prime i}x^{\prime j}\right)ds\\ = & \frac{1}{2}\int_{s_{0}}^{s_{1}}\left[\partial_{k}g_{ij}x^{\prime i}x^{\prime j}\delta x^{k}-\frac{d}{ds}\left(g_{ij}x^{\prime j}\right)\delta x^{i}-\frac{d}{ds}\left(g_{ij}x^{\prime i}\right)\delta x^{j}\right]ds\\ \; & +\underset{\mathrm{boundary term ℬ}}{\underset{︸}{\frac{1}{2}{\left.\left(g_{ij}\delta x^{i}x^{\prime j}+g_{ij}x^{\prime i}\delta x^{j}\right)\right|}_{s_{0}}^{s_{1}}}}\\ = & \frac{1}{2}\int_{s_{0}}^{s_{1}}\left(\partial_{k}g_{ij}x^{\prime i}x^{\prime j}-\partial_{i}g_{kj}x^{\prime j}x^{\prime i}-\partial_{j}g_{ik}x^{\prime i}x^{\prime j}-2g_{kl}x^{\prime \prime l}\right)\delta x^{k}ds+B \end{array}$$

which vanishes for any $\delta x^{k}$. The geodesic equation becomes

(A25) $$x^{\prime \prime l}+\underset{\Gamma {}_{ij}^{l}}{\underset{︸}{\frac{1}{2}g^{kl}\left(\partial_{i}g_{kj}+\partial_{j}g_{ik}-\partial_{k}g_{ij}\right)}}x^{\prime i}x^{\prime j}=0$$

where $\Gamma {}_{ij}^{l}$ are the Christoffel symbols. Now we compute these components (noticing that $\Gamma {}_{ij}^{l}=\Gamma {}_{ji}^{l}$):

(A26) $$\begin{array}{c} \left[\begin{array}{c} \Gamma^{1}\\ \Gamma^{2} \end{array}\right]=\frac{1}{2(EG-F^{2})}\left[\begin{array}{cc} G\partial_{1}E-2F\partial_{1}F+F\partial_{2}E & G\partial_{2}E-F\partial_{1}G\\ G\partial_{2}E-F\partial_{1}G & 2G\partial_{2}F-G\partial_{1}G-F\partial_{2}G\\ 2E\partial_{1}F-E\partial_{2}E-F\partial_{1}E & E\partial_{1}G-F\partial_{2}E\\ E\partial_{1}G-F\partial_{2}E & E\partial_{2}G-2F\partial_{2}F+F\partial_{1}G \end{array}\right] \end{array}$$

After computations we have

(A27) $$\Gamma {}_{ij}^{k}=\frac{\partial_{ij}\Delta \partial_{k}\Delta }{1+{\left(\partial_{1}\Delta \right)}^{2}+{\left(\partial_{2}\Delta \right)}^{2}}$$

Hence, the geodesic equation is given by

(A28) $$\frac{d^{2}}{ds^{2}}\left[\begin{array}{c} x^{1}\\ x^{2} \end{array}\right]=-\frac{1}{\sqrt{g}}\left(b:\frac{dx}{ds}⊗\frac{dx}{ds}\right)\left[\begin{array}{c} \partial_{1}\Delta \\ \partial_{2}\Delta \end{array}\right]$$

### Appendix A.5. The First Fundamental Form

In this appendix we consider the first fundamental form, from which we obtain the steepest descent curves and level set curves in Section 3.2.4.

The first fundamental form g is a (0,2)-tensor, which induces an inner product between two vectors v and w belonging to the tangent space $T_{p}\Delta$ by ${\langle v,w\rangle }_{g}=g:vw$, and we usually neglect g.

The first fundamental form g gives a good approximation of the distance between two very close points on the surface, thus the infinitesimal distance $ds^{2}=g_{\mu \nu }dx^{\mu }⊗dx^{\nu }$ and the volume form $\mathrm{vol}=\sqrt{\left|\det g\right|}dx^{1}\wedge \ldots dx^{n}$.

We can also find the analytic solutions of the two eigenvector fields of g by

(A29) $$\begin{array}{cc} r_{1}:\; & \ln\left|\frac{\tan S\pi }{\cos S\pi }\right|+\chi =\frac{\pi^{2}}{4}C\\ r_{2}:\; & 3-\cos2S\pi +\chi =\sqrt[{4}]{C} \end{array}$$

where χ is a constant representing a family of curves. The first family is the curves of steepest descent and the second family the level set curves.

## Appendix B. Variational Method

In this appendix, we provide a detailed derivation of the variation of mean curvature. The results in this appendix are used without further explanation in Equation (5) to Equation (7), as well as Table 2 and Table 3.

We adopt notation $e_{i}:=\partial_{i}$ as the tangent vector to not confuse with the partial derivative.

### Appendix B.1. Background

The variations of the following results can be found in many literatures [68] and we only use the result here.

The variation of tangent vector $\partial_{i}$ due to $\dot{r}=U^{i}\partial_{i}+Vk$ is

(A30) $$\begin{array}{c} \frac{\delta e_{i}}{\delta t}=\left(\nabla_{i}U^{k}-Vb_{i}^{k}\right)e_{k}+\left(U^{k}b_{ki}+\nabla_{i}V\right)k \end{array}$$

The variation of the first fundamental form using the Lie derivative L is

(A31) $$\begin{array}{c} \frac{\delta g_{ij}}{\delta t}=L_{U}g_{ij}-2Vb_{ij}+\frac{1}{\delta t}\delta \delta g_{ij} \end{array}$$

The variation of the second fundamental form is

(A32) $$\begin{array}{c} \frac{\delta b_{ij}}{\delta t}=\nabla_{i}\nabla_{j}V-b_{ik}b_{j}^{k}V+L_{U}b_{ij}+\frac{1}{\delta t}\delta \delta b_{ij} \end{array}$$

### Appendix B.2. Variation of Curvatures

The tensorial method in differential geometry benefits the computations of the curvatures. A tensor contraction by dropping the O(2) term yields

(A33) $$\begin{array}{cc} \frac{\delta H}{\delta t}= & \frac{1}{2\delta t}\delta \left(g^{ij}b_{ij}\right)=\frac{1}{2\delta t}\left(\delta g^{ij}b_{ij}+g^{ij}\delta b_{ij}\right)\\ = & \frac{1}{2}\left[-\left(\nabla^{i}U^{j}+\nabla^{j}U^{i}-2Vb^{ij}\right)b_{ij}+g^{ij}\left(\nabla_{i}\nabla_{j}V-b_{ij}b_{j}^{k}V+U^{k}\nabla_{k}b_{ij}+b_{kj}\nabla_{i}U^{k}+b_{ik}\nabla_{j}U^{k}\right)\right]\\ = & -\frac{1}{2}\left(\nabla^{i}U^{i}+\nabla^{j}U^{i}\right)b_{ij}+V\left(4H^{2}-2K\right)+\frac{1}{2}\left[\nabla^{2}V-\left(4H^{2}-2K\right)V+2U^{i}\nabla_{i}H+2b_{i}^{k}\nabla_{k}U^{i}\right]\\ = & V\underset{H^{2}+D^{2}}{\underset{︸}{\left(2H^{2}-K\right)}}+\underset{U\cdot \nabla_{s}H}{\underset{︸}{U^{i}\nabla_{i}H}}+\frac{1}{2}\nabla_{s}^{2}V \end{array}$$

The variation of the deviatoric curvature can be computed in a similar way [47,48]

(A34) $$\frac{\delta D}{\delta t}=U\cdot \nabla_{s}D+2HDV+\frac{1}{2}q:\nabla_{s}\nabla_{s}V$$

Hence, the variation of Gaussian curvature can be computed by $\dot{K}:=\delta_{t}\left(H^{2}-D^{2}\right)=2H\dot{H}-2D\dot{D}$, reducing to

(A35) $$\begin{array}{c} \frac{\delta K}{\delta t}=U\cdot \nabla_{s}K+2HKV+H\nabla_{s}^{2}V-Dq:\nabla_{s}\nabla_{s}V \end{array}$$

And the variation of the Casorati curvature can be obtained from $C^{2}=H^{2}+D^{2}$

(A36) $$\begin{array}{c} \frac{\delta C}{\delta t}=\frac{1}{2C}\left[2VH\left(H^{2}+3D^{2}\right)+U\cdot \nabla_{s}C^{2}+H\nabla_{s}^{2}V+q:\nabla_{s}\nabla_{s}V\right] \end{array}$$

Along with the variation of the shape parameter $\dot{S}$

(A37) $$\begin{array}{c} \frac{\delta S}{\delta t}=\frac{2}{\pi C^{2}}\left[-VDK+U\cdot \left(D\nabla_{s}H-H\nabla_{s}D\right)+\frac{1}{2}\left(D\nabla_{s}^{2}V-Hq:\nabla_{s}\nabla_{s}V\right)\right] \end{array}$$

## Appendix C. Average Entropy Production Rate $\bar{\Delta }$

The goal in this appendix is to provide a more detailed discussion about Figure 11 and Figure 12. We first compute the average entropy production rate in terms of *C* and *S* along the astigmatic flow A(m), which gives the relation between *S* and *C* such that

(A38) $$C=\frac{m\cos(\frac{S\pi }{2})}{2\cos^{2}\left(\frac{S\pi }{2}\right)-1}=\frac{m\cos(\frac{S\pi }{2})}{\cos S\pi }\;\mathrm{and}\;\frac{dC}{dS}=\frac{m\pi \sin\left(\frac{S\pi }{2}\right)\left[2\cos^{2}\left(\frac{S\pi }{2}\right)+1\right]}{2\cos^{2}S\pi }$$

The shape-average ${\bar{\Delta }}_{S}$ can be computed analytically,

(A39) $$\begin{array}{cc} {\bar{\Delta }}_{S}= & \frac{1}{S_{f}-S_{0}}\int_{A}\Delta dS=\frac{1}{S_{f}-S_{0}}\int_{A}C^{4}\left(1+\sin^{2}S\pi \right)dS\\ = & \frac{1}{S_{f}-S_{0}}\int_{S_{0}}^{S_{f}}dS\frac{m^{4}}{\cos^{4}dS\pi }\left[\left(1+\sin^{2}S\pi \right)\cos^{4}\left(\frac{S\pi }{2}\right)\right]\\ = & \frac{m^{4}}{S_{f}-S_{0}}{\left.\left[\underset{\mathrm{Sh}(S)}{\underset{︸}{\frac{1}{48\pi }\sec^{3}S\pi \left(15\sin S\pi +12\sin2S\pi +7\sin3S\pi -9S\pi \cos S\pi -3S\pi \cos3S\pi \right)}}\right]\right|}_{S_{0}}^{S_{f}} \end{array}$$

where we neglect the constant and define function Sh(S). If m<0, then $S_{f}=\pm 1$ and $0.5<|S_{0}|<1$. If m>0, then $S_{f}=0$ and $|S_{0}|<0.5$,

(A40) $$\begin{array}{c} {\bar{\Delta }}_{S}\left(m<0\right)=\frac{m^{4}}{\mp 1-S_{0}}\left(\mathrm{Sh}\left(\mp 1\right)-\mathrm{Sh}\left(S_{0}\right)\right)\\ {\bar{\Delta }}_{S}\left(m>0\right)=-\frac{m^{4}}{S_{0}}\left(\mathrm{Sh}\left(0\right)-\mathrm{Sh}\left(S_{0}\right)\right) \end{array}$$

where

(A41) $$\mathrm{Sh}\left(\mp 1\right)=\pm \frac{1}{4},\;\mathrm{Sh}\left(0\right)=0$$

The curvedness-average ${\bar{\Delta }}_{C}$ can be computed in the same way if $C_{0}$ is givenThe shape-average ${\bar{\Delta }}_{S}$ can be computed analytically,

(A42) $$\begin{array}{cc} {\bar{\Delta }}_{C}= & \frac{1}{C_{f}-C_{0}}\int_{A}\Delta dC=\frac{1}{C_{f}-C_{0}}\int_{A}C^{4}\left(2-\cos^{2}S\pi \right)dC\\ = & \frac{1}{C_{f}-C_{0}}\int_{C_{0}}^{C_{f}}dCC^{4}\left[2-\frac{m^{2}{\left(\sqrt{8C^{2}+m^{2}}+m\right)}^{2}}{16C^{4}}\right]\\ = & \frac{1}{C_{f}-C_{0}}{\left.\left[\underset{\mathrm{Cur}(C,m)}{\underset{︸}{\frac{2}{5}C^{5}-\frac{m^{2}}{6}C^{3}-\frac{m^{3}}{16}C\sqrt{8C^{2}+m^{2}}-\frac{m^{4}}{8}C-\frac{m^{5}}{32\sqrt{2}}\ln\left(\sqrt{2}\sqrt{8C^{2}+m^{2}}+4C\right)}}\right]\right|}_{C_{0}}^{C_{f}} \end{array}$$

where the constant is neglected, and the function Cur(C,m) is defined. Here we should notice that the function Sh(S) and Cur(C,m) are different, where *m* can be decoupled from Sh while *C* and *m* are intertwined in Cur. $C_{0}\left(C_{f}\right)$ is determined by giving $S_{0}\left(S_{f}\right)$ and *m*. Similarly if m<0, then $C_{f}=0$ and if m>0 then $C_{f}=m$.

(A43) $$\begin{array}{c} {\bar{\Delta }}_{C}\left(m<0\right)=-\frac{1}{C_{0}}\left(\mathrm{Cur}\left(0,m\right)-\mathrm{Cur}\left(C_{0},m\right)\right)\\ {\bar{\Delta }}_{C}\left(m>0\right)=\frac{1}{m-C_{0}}\left(\mathrm{Cur}\left(m,m\right)-\mathrm{Cur}\left(C_{0},m\right)\right) \end{array}$$

where

(A44) $$\begin{array}{c} \mathrm{Cur}\left(0,m\right)=-\frac{m^{5}}{32\sqrt{2}}\ln(\sqrt{2}\left|m|\right)\\ \mathrm{Cur}\left(m,m\right)=m^{5}\left(-\frac{19}{240}-\frac{1}{32\sqrt{2}}\ln\left(\left(3\sqrt{2}+4\right)m\right)\right) \end{array}$$

We can regard ${\bar{\Delta }}_{C}$ and ${\bar{\Delta }}_{S}$ in Section 3.3.3 in a geometric view if we process the following operation

(A45) $${\bar{\Delta }}_{C}=\frac{1}{C_{f}-C_{0}}\int_{A}\Delta dC=\frac{1}{\left(C_{f}-C_{0}\right)\left(S_{f}-S_{0}\right)}\int_{S_{0}}^{S_{f}}dS\int_{A}\Delta dC=\frac{1}{\Omega }{\;\int \int \;}_{\Omega }{\Delta |}_{\Omega_{C}}dS\wedge dC=\frac{V_{C}}{\Omega }$$

A similar construction of ${\bar{\Delta }}_{S}$ yields

(A46) $${\bar{\Delta }}_{S}=\frac{1}{\Omega }{\;\int \int \;}_{\Omega }{\Delta |}_{\Omega_{S}}dS\wedge dC=\frac{V_{S}}{\Omega }$$

Figure A1 demonstrates the geometric interpretation of Equations (A45) and (A46). The invariant *m* determines (here m<0) an astigmatic flow ${\Delta |}_{A}$ shown as the 3-D magenta curve along Δ surface in Figure A1a. $S_{0}$ determines the two endpoints of this curve. A red triangular prism can be uniquely determined with one curved surface (zero Gaussian curvature) according to the position of ${\Delta |}_{A}$ shown in Figure A1, where the curve is obtained from projecting ${\Delta |}_{A}$ onto (C,Δ)-plane. This curved triangular prism is denoted by $V_{C}$. The projection of the $V_{C}$ onto (C,S)-plane is Ω, and the projection onto (C,Δ)-plane is $\Omega_{C}$. Similarly, we can construct another blue triangular prism with one curved surface where the curve is obtained from projecting ${\Delta |}_{A}$ onto (S,Δ)-plane, explained as Figure A1b. Therefore, Equations (A45) and (A46) elucidate the fact that the average entropy production is just the volume of the curved triangular prism divided by the bottom area Ω.

It can be verified that the shape average is no greater than curvedness average, they only agree when the initial shape is the sphere or the saddle. To explain this, we just need to compare the volume of the red and blue curved triangular prisms and show that $V_{C}\geq V_{S}$ in Figure A1 by Equations (A45) and (A46). Stokes’ theorem transferred the integral over Ω to the closed integral over the boundary ∂Ω (see the middle top subfigure Figure A1).

(A47) $${\;\int \int \;}_{\Omega }\underset{d\Gamma_{C}}{\underset{︸}{{\mathrm{Proj}}_{\Omega_{C}}\left(\Delta {|}_{A}\right)dC\wedge dS}}=\oint_{\partial \Omega }\Gamma_{C}∼\mathrm{Cur}>\mathrm{Sh}∼\oint_{\partial \Omega }\Gamma_{S}={\;\int \int \;}_{\Omega }\underset{d\Gamma_{S}}{\underset{︸}{{\mathrm{Proj}}_{\Omega_{S}}\left(\Delta {|}_{A}\right)dC\wedge dS}}$$

where ${\mathrm{Proj}}_{\Omega_{C(S)}}\left(⋆\right)$ is the projection operator to the plane $\Omega_{C(S)}$ and $d\Gamma_{C(S)}$ is the exact differential 2-form. In addition, the volume $V_{C(S)}$ rises faster than the bottom area Ω with the enhancement of |m|.

Figure A1 also provides another view to explain the generality of ${\bar{\Delta }}_{C}$. If ${\Delta |}_{A}$ follows $S_{0}=-1$ or $S_{0}=-1/2$, $\Omega_{C}$ is smoothly changed but $\Omega_{S}$ disappeared as we can see from Figure A1b. Hence Equation (A46) is not defined while Equation (A45) still provides a good method to average the thermodynamic surface.
